# Spatial inequalities in cardiovascular health: a cross-sectional study with small-area health insurance claims and individual-level primary care data in Belgium

**DOI:** 10.1186/s12889-026-27365-6

**Published:** 2026-04-23

**Authors:** Arne Janssens, Sumbul Hashmi, Anne-Laure Budts, Lorenz Van der Linden, Wies Kestens, Spencer Marcinko, Chiel Hex, Thomas Neyens, Ellie D’Hondt, Caroline Van Cauwelaert, Bert Vaes

**Affiliations:** 1 Academic Center for General Practice, Department of Public Health and Primary Care, KU Leuven, 7001, Kapucijnenvoer 7, Leuven, B-3000 Belgium; 2 EPCON, Lange Gasthuisstraat 29/31, Antwerp, B-2000 Belgium; 3 Clinical Pharmacology and Pharmacotherapy, Department of Pharmaceutical and Pharmacological Sciences, KU Leuven, B-3000 Leuven, Belgium; 4 Pharmacy Department, University Hospitals Leuven, B-3000 Leuven, Belgium; 5 Mutualités Libres/Onafhankelijke Ziekenfondsen (MLOZ), Lenniksebaan 788A, Anderlecht, B-1070 Belgium; 6 Department of Healthcare and Ethics, Faculty of Medicine and Life Sciences, Hasselt University, Agoralaan, Diepenbeek, B-3590 Belgium; 7 L-BioStat, Department of Public Health and Primary Care, KU Leuven, Kapucijnenvoer 35, Leuven, B-3000 Belgium; 8 I-BioStat, Data Science Institute, Hasselt University, Martelarenlaan 42, Hasselt, B- 3500 Belgium; 9 Cascador Health - Fidux Health, 1A, Kardinaal Mercierlaan, Merelbeke-Melle, 9090 Belgium

**Keywords:** Atherosclerotic cardiovascular disease, Socioeconomic vulnerability, Health inequalities, Spatial epidemiology, Primary care data, Health insurance data, Public health surveillance, Geographic disparities, Small-area data

## Abstract

**Aims:**

Atherosclerotic cardiovascular disease (ASCVD) continues to pose a growing burden driven by aging, comorbidities, and socioeconomic vulnerability. Yet, the regional distribution of these disparities remains poorly characterized, partly because no single dataset provides comprehensive ASCVD surveillance data. To address this gap, we combined insights from small-area health insurance claims and individual-level primary care data to assess how socioeconomic vulnerability relates to ASCVD indicators across regional contexts in Belgium.

**Methods:**

We conducted a cross-sectional study using two complementary data sources: (i) small-area ASCVD indicators from a nationwide health insurance organization and (ii) individual-level clinical diagnoses from a general practice (GP) registry in Flanders. A multidimensional small-area socioeconomic vulnerability index was constructed using established methodology. Associations were examined using Spearman correlation, Mann-Whitney U tests, bootstrapping, and hierarchical logistic regression models.

**Results:**

Both small-area health insurance claims data and small-area predictions derived from individual-level GP registry data revealed regional disparities in ASCVD prevalence that overlapped with patterns of socioeconomic vulnerability. Socioeconomic vulnerability was positively correlated with ASCVD prevalence at both small-area and individual levels. In the claims dataset, the median ASCVD prevalence was 1.57 times higher (95% CI: 1.46–1.65) in high-vulnerability areas compared with low-vulnerability areas. In the GP registry, overall ASCVD prevalence was 6.46%, with marked disparities by age, sex, socioeconomic status, and lipid-lowering medication. Increased compensation, a proxy for individual-level socioeconomic vulnerability, was associated with higher ASCVD prevalence, particularly among individuals not receiving lipid-lowering therapy. Small-area risk estimates of increased compensation derived from the GP registry showed moderate agreement with the multidimensional vulnerability index. An accompanying geoportal visualizes small-area data, enabling stakeholders to identify high-vulnerability areas and support targeted public health interventions.

**Conclusion:**

Combining small-area health insurance claims with individual-level primary care data provides a more comprehensive framework for cardiovascular health surveillance, revealing socioeconomic disparities that are not fully captured by either dataset alone. The agreement between the findings from these data sources strengthens confidence in the observed spatial patterns and highlights regional inequalities in indicators of ASCVD prevalence across Belgium, underscoring the value of integrated data approaches for public health monitoring and the need for localized public health interventions addressing both area-level and individual-level social determinants of health.

**Supplementary Information:**

The online version contains supplementary material available at 10.1186/s12889-026-27365-6.

## Introduction

Cardiovascular disease (CVD) remains the leading cause of death globally, accounting for approximately one-third of all deaths and placing a substantial burden on healthcare systems [1, 2]. Atherosclerotic cardiovascular disease (ASCVD) represents around 85% of all CVD cases and continues to exert a growing societal impact due to aging populations, increased prevalence of diabetes, hypertension, obesity as well as persistent health inequities in access to preventive care and treatment [3, 4].

In Belgium, ASCVD accounts for approximately one-fourth of annual deaths [3], with substantial regional disparities in ASCVD-related mortality rates between the North (Flanders) and South (Wallonia) regions [5]. These differences are likely influenced by variations in local socioeconomic conditions [6, 7], as socioeconomic status (SES) is consistently linked with cardiovascular health and ASCVD outcomes [8, 9]. To describe the SES of a population, the World Health Organization (WHO) defines the social determinants of health (SDOH), which “are the conditions in which people are born, grow, live, work and age, and people’s access to power, money and resources“ [10]. Both individual-level and area-level SDOH (e.g., income, education, employment) contribute to disparities in ASCVD outcomes [11].

SES is inherently multifactorial but often approximated by unidimensional proxies such as income, employment, or education [8]. While these measures may inadequately capture the multifactorial nature of SES and its relation with ASCVD risk [8], the social and physical environment of the area where people live may influence cardiovascular risk, in addition to individual socioeconomic characteristics [12]. Multidimensional, composite area-level indices that integrate several SES domains have therefore been developed to characterise these community-level characteristics [13–16]. These indices have demonstrated associations with ASCVD risk in multiple countries [7, 17]. They may enable a better understanding of the spatial relationship between socioeconomic vulnerability and ASCVD, which is essential for designing geographically targeted and data-driven prevention strategies.

However, in Belgium, several limitations hinder these analyses. National ASCVD prevalence data are outdated, and the most recent Belgian Index of Multiple Deprivation (BIMD) dates back to 2011 [16]. Moreover, no study has yet examined spatial association between socioeconomic vulnerability and ASCVD risk. Finally, it remains unclear whether existing datasets are meaningfully aligned to support such analyses.

To address these gaps, the study aims to: (i) estimate the small-area prevalence of ASCVD in Belgium using two distinct data sources; (ii) construct an updated, multidimensional area-level deprivation index; (iii) assess associations between socioeconomic vulnerability and ASCVD risk, and their regional variations; (iv) compare the findings across the two datasets to evaluate consistency and identify potential discrepancies. Additionally, we developed a customized geoportal for mapping relevant data layers, enabling stakeholders to visualize regional disparities intuitively.

## Methods

### Study design and data sources

We conducted a retrospective, observational study to assess the correlation between socioeconomic vulnerability and ASCVD prevalence for the year 2023. ASCVD data were obtained from two complementary sources: (1) small-area administrative data from the health insurance fund, Multualités Libres - Onafhankelijke Ziekenfondsen (MLOZ), and (2) individual-level clinical data from the Intego-II primary care database [18].

#### MLOZ: small-area claims data

In Belgium, health insurance is required for all registered residents and provided through one of the seven insurance funds. MLOZ is the third-largest fund, covering approximately 18% of the Belgian population. Small-area data were provided at the level of municipality and statistical sector, the latter being the smallest geographical unit in Belgium [19].

The data underwent a privacy risk assessment, performed by an independent third party Cascador Health, to minimize re-identification risk and ensure GDPR-compliant reporting. They applied a k-anonymity rule and suppressed strata where the count was smaller than three (i.e., small cells), and additionally flagged strata with a denominator smaller than 25 as potentially statistically unstable.

Following the exclusion of small cells for privacy reasons, the dataset encompassed 20% of the Belgian statistical sectors, distributed heterogeneously across the country, which limited their suitability for the present analyses. For this reason, we restricted our analyses to the municipality level, where coverage was near-complete. In 2023, Belgium comprised 581 municipalities. Data included ASCVD indicators related to cardiovascular interventions and the dispensation of reimbursed cardiovascular drugs per geographical unit, detailed in the study population and data variables section. For simplicity, this dataset will be referred to as ‘small-area claims data’ throughout this paper.

#### Intego: GP registry data

The Intego-II primary care database is a general practice-based morbidity registration network coordinated by the Department of Public Health and Primary Care of KU Leuven. It contains pseudonymized patient-level health data from general practitioners (GPs) across Flanders, covering 7.35% of the Flemish population [18]. Currently, the network comprises 135 active general practice centers using Corilus CareConnect^®^ software [18]. Data are automatically collected during routine care and include diagnoses, sociodemographic information, prescriptions, vaccinations, laboratory results and biomedical parameters, such as blood pressure, height and weight. The registry is hosted on the Healthdata platform [20]. For simplicity, this dataset will be referred to as ‘GP registry data’ throughout this paper.

#### Publicly available datasets

Publicly available datasets were used to develop a multidimensional socioeconomic vulnerability index similar to the Belgian Index of Multiple Deprivation (BIMD) [3] and to predict small-area ASCVD prevalence in Flanders from the GP registry model. Data were sourced from the Intermutualistic Agency (IMA), Statbel, and the Federal Police of Belgium. IMA aggregates patient data from all seven Belgian health insurance funds on a single platform and provides demographic, socio-economic and healthcare reimbursement data. Aggregated data are publicly accessible from https://atlas.ima-aim.be/databanken. Statbel, the Belgian statistical office, provides population data at https://statbel.fgov.be. Crime statistics were obtained from the federal police at www.politie.be.

### Study population and data variables

#### Small-area claims data

The study population included all individuals who were members of MLOZ at any point in 2023 and officially resided in Belgium. More detailed information about the demographic distribution with respect to the Belgian population and geographic coverage is included in Additional file 1, Table A1_1, and Figure A1_1, respectively. The data analysed included proportions at the municipality level, in line with standard small-cell risk mitigation strategies to protect individual privacy.

Two categories of indicators were defined: interventional and pharmacological indicators, which are detailed in Additional files 2 and 3, respectively. Interventional indicators were selected as the primary proxy for ASCVD prevalence because they indicate established disease. In contrast, pharmacological indicators in the claims data are broad and non-specific, as lipid-lowering and antihypertensive medications are prescribed for both primary and secondary prevention and for conditions unrelated to ASCVD, while also failing to capture individuals with established ASCVD who are untreated or not recorded as chronic users. Interventional indicators included ASCVD-related surgical procedures and interventions, captured using nomenclature codes, defined by the National Institute for Health and Disability Insurance and validated by three clinical experts (Additional file 2). Proportions were calculated for the overall study population and stratified by age (< 50 years vs. ≥50 years), and by increased healthcare reimbursement status (proxy for socio-economic vulnerability). Age was dichotomized at 50 years, reflecting a pragmatic threshold at which ASCVD risk increases markedly, and representing the finest age stratification feasible at the municipality level, given privacy-related small-cell constraints. The numerator was the number of individuals with any ASCVD-related intervention between January 1, 2013, and December 31, 2023 per stratum, and the denominator was the corresponding number of individuals in the study population per stratum.

Pharmacological indicators reflect chronic use of medications with lipid-modifying agents identified by Anatomical Therapeutic Chemical Classification System (ATC) codes starting with “C10” (Additional file 3). Chronic use is pragmatically defined as at least 80 daily defined doses per year [21]. The administrative database contained only information on dispensed and reimbursed medications. Proportions were obtained for medications containing statins only and for those including statins and other lipid-lowering medication. Numerators were the number of individuals in the study population with chronic use in 2023, and denominators were the number of individuals in the study population. These proportions were also stratified by age group.

#### GP registry data

The GP registry’s study population included all patients who had at least one clinical encounter with a GP in 2023, i.e., the yearly contact group of that year. Patients who died in 2023 or resided in municipalities outside of Flanders were excluded from the analysis. More detailed information about the demographic distribution with respect to the Flemish population is included in Table A1_2 in Additional file 1.

For each patient, we extracted the following variables: ASCVD status, defined as a confirmed diagnosis of ASCVD based on International Codes of Primary Care (version 2; ICPC-2) codes recorded before December 31, 2023 (Additional file 4); medication use, indicated by a prescription for lipid-lowering medications in 2023 (Additional file 3); sex (male or female); age (categorized as < 50 and ≥ 50, using the same cut-off as in the small-area claims data to ensure comparability across datasets); increased compensation status, reflecting whether the patient receives enhanced reimbursement for healthcare as a proxy for socioeconomic vulnerability; municipality, representing the patient’s place of residence; and an identifier for visited practice. Increased compensation status (in Dutch: “verhoogde tegemoetkoming”) is a categorical Belgian health insurance benefit granted to socially vulnerable groups, including low-income households and recipients of social assistance, entitling them to a higher reimbursement rate for healthcare costs (see Additional file 5).

Compared with the small-area claims data, which reflected chronic use of dispensed and reimbursed lipid-lowering medications, the GP registry captured prescribed lipid-lowering medications at the individual patient level. ASCVD status in the GP dataset was based on clinically confirmed diagnoses recorded by GPs, whereas in the small-area claims data, ASCVD was inferred indirectly through diagnostic codes associated with reimbursed interventions. The GP registry thus allowed for individual-level analyses, but only at the municipality level in Flanders, while the claims data enabled analyses across the whole of Belgium.

### Statistical analysis

We followed a four-step analysis plan to: (i) estimate the small-area prevalence of ASCVD, (ii) compute a multidimensional socioeconomic vulnerability index, (iii) evaluate the association between socioeconomic vulnerability and ASCVD prevalence, and (iv) compare results between the small-area claims and GP registry datasets. Each step is described in detail below.

#### Step 1: Estimation of ASCVD prevalence

Interventional indicators were used as a primary proxy for ASCVD prevalence in the small-area claims dataset, while pharmacological indicators served as an additional, more illustrative proxy. The GP registry data provided patient-level information and enabled estimation of ASCVD prevalence at the regional level (Flanders) [18], but did not allow reliable comparisons between municipalities due to imbalanced coverage.

To estimate small-area, municipality-level, ASCVD prevalence from the GP registry data, we first modelled the relationship between ASCVD and key covariates and then predicted through this model municipality-level prevalences using publicly available population data from IMA, which provided data on the number of inhabitants per municipality and per strata of age group, sex, and increased compensation. We applied a binomial generalized linear mixed model (GLMM) that included the following fixed effects: age group (< 50, ≥ 50), sex, increased compensation, medication use, and an interaction term between increased compensation and medication use. Random intercepts to account for the hierarchical nature. The model is detailed in Additional file 6.

Because medication use was not available in the IMA data, we estimated medication use proportions for each stratum of age, sex, and increased compensation based on the GP registry data. Using the fixed effects from the GLMM together with these estimated medication use proportions, we predicted ASCVD prevalence at the municipality-level for the IMA population. To facilitate these predictions, we used a model without random effects. The municipality-level ASCVD prevalence predictions obtained were then used to generate a prevalence map for Flanders (see Additional file 6).

#### Step 2: Computation of the multidimensional socioeconomic vulnerability index

As the most recent Belgian Index of Multiple Deprivation (BIMD) dated from 2011, we constructed a current multidimensional small-area socioeconomic vulnerability index, following the same methodological framework with six domains of deprivation: income, employment, education, housing, health, and crime. Adaptations were made to specific indicators within these domains based on data availability (see Additional file 7). Individual indicators were initially aggregated into six domain-specific scores, which were subsequently combined to derive an overall vulnerability score. At both stages, the same procedure was applied: inputs were standardized through ranking and scaling, and then summed according to the weights specified in the original publication. The range of the vulnerability index varies between 1 and 100, from least deprived [1] to most deprived (100).

#### Step 3: Assess associations between ASCVD prevalence and socioeconomic vulnerability

We assessed the association between ASCVD prevalence and socioeconomic vulnerability using Spearman correlations and the Mann-Whitney U test on both small-area claims data and model-based predictions from the GP registry. The vulnerability index was divided into quintiles, and differences between the most (top quintile) and least (bottom quintile) vulnerable municipalities were tested with the Mann-Whitney U test. A nonparametric bootstrap procedure was used to estimate the relative difference (multiplicative effect) between the median ASCVD ratios of high- and low-vulnerability municipalities, yielding point estimates and 95% bootstrap confidence intervals. Individual-level associations from the GP registry were evaluated using the GLMM described in “Step 1” and in Additional file 6.

#### Step 4: Compare findings from small-area claims data and GP registry data

We compared ASCVD prevalence and socioeconomic vulnerability measures from the two data sources by using Spearman’s rank correlation. In the GP registry data, SES was approximated by the indicator of receiving increased compensation. To examine whether this SES indicator reflects similar spatial patterns as the vulnerability index, we estimated municipality-level odds ratios of receiving increased compensation using a Binomial GLMM accounting for sex, age, and practice. The model is detailed in Additional file 6.

#### Geoportal

ASCVD prevalence indicators of the small-area claims data and the vulnerability index were visualized as choropleth maps on a customized Geoportal hosted on Amazon Web Services, Inc. server. The indicators were made available as countrywide spatial data layers aggregated to municipality and statistical sector level. Data from the GP registry was excluded from this visualization because it does not cover the whole country but only the Flanders region of Belgium. The portal can be accessed at https://ascvd-heatmap.epcon.ai/.

#### Software and analysis

The small-area claims data were analysed in Python (version 3.12.5). All GP registry analyses were conducted in R (version 4.0.5). GP registry models were fitted using the R-INLA package with Integrated Nested Laplace Approximation (INLA) for approximate Bayesian inference. Model selection was based on Deviance Information Criterion (DIC) and Watanabe-Akaike Information Criterion (WAIC). After a prior sensitivity check, default INLA priors were applied for the precisions of the two random effects ($\tau\:\sim\mathrm{Gamma}\left(\mathrm{0,0.00005}\right)$). Parameter estimates were reported with 95% credible intervals. Spearman correlations and Mann-Whitney U tests were performed using a significance level of 0.05.

## Results

### Study population characteristics

The small-area claims database comprised 2,291,621 clients across the country. This population included 809,238 people who were 50 years and older (≥ 50), and 1,482,383 people younger than 50 years (< 50), with an equal distribution of males and females. Compared with the general Belgian population, individuals receiving increased compensation were underrepresented in the MLOZ dataset (14% vs. 20%; Additional file 1, Table A1_1). Geographic coverage of the MLOZ population across Belgian municipalities is shown in Figure A1_1 in Additional file 1. Table 1 provides a descriptive view of the primary variables of interest expressed as proportions aggregated to the municipality level.

**Table 1:** Descriptive statistics of three indicators for ASCVD prevalence from the small-area claims data, expressed as %

Indicator (%)	Subgroups	Number of municipalities	Mean (sd)	Median (IQR)
ASCVD intervention	Overall	574	2.12 (0.71)	1.99 (0.85)
	Increased compensation	422	0.45 (0.30)	0.35 (0.39)
	No increased compensation	424	1.74 (0.56)	1.68 (0.55)
	≥ 50 years	574	5.48 (1.37)	5.38 (1.79)
	< 50 years	203	0.20 (0.13)	0.18 (0.13)
Statin	Overall	580	10.18 (2.58)	9.89 (3.16)
	≥ 50 years or older	579	25.5 (4.46)	25.32 (5.34)
	< 50 years	527	1.30 (0.51)	1.24 (0.6)
Other lipid-lowering medication	Overall	576	4.26 (1.45)	4.00 (2)
	≥ 50 years	575	10.48 (2.76)	10.05 (4.03)
	< 50 years	451	0.66 (0.31)	0.58 (0.37)

The GP registry’s dataset started with 428,170 adult patients in the yearly contact group of 2023. After excluding two patients without municipality information and 3,561 patients residing outside of Flanders, the final study population consisted of 424,607 patients, representing 99.17% of the original yearly contact group. Compared with the general Flemish population, the GP registry study population shows similar distributions by age, sex, and increased compensation status (Additional file 1, Table A1_2). An overview of the study population characteristics can be found in Table 2.

**Table 2:** Characteristics of the study population of the GP registry dataset

Variable	Subgroup	Number of patients (%)	Number of prevalent ASCVD cases (%)
Total		424,607	27,441 (6.46%)
Sex	Female	226,252 (53.29%)	12,144 (5.37%)
	Male	198,355 (46.71%)	15,297 (7.71%)
Age group	Under 50	243,371 (57.32%)	2,313 (0.95%)
	Over 50	181.236 (42.68%)	25,128 (13.86%)
Increased compensation	Yes	63.809 (15.03%)	6,246 (9.79%)
	No	360.798 (84.97%)	21,195 (5.87%)
Medication use	Yes	71,934 (16.94%)	17,604 (24.47%)
	No	352,673 (83.06%)	9,837 (2.79%)

### Prevalence of ASCVD

Table 1 presents descriptive statistics of the small-area claims data at municipality level concerning three indicators for ASCVD prevalence. For each of the three indicators, it includes the number of municipalities for which we received a value after excluding small cells, along with the mean, standard deviation, median, and the quartiles over the corresponding municipalities. For example, the mean ASCVD intervention proportion across 574 municipalities was 2.12%, with the median at 1.99%. Similar numbers are shown for the stratified ASCVD intervention, statin, and other lipid-lowering medication proportions.

**Fig. 1:**
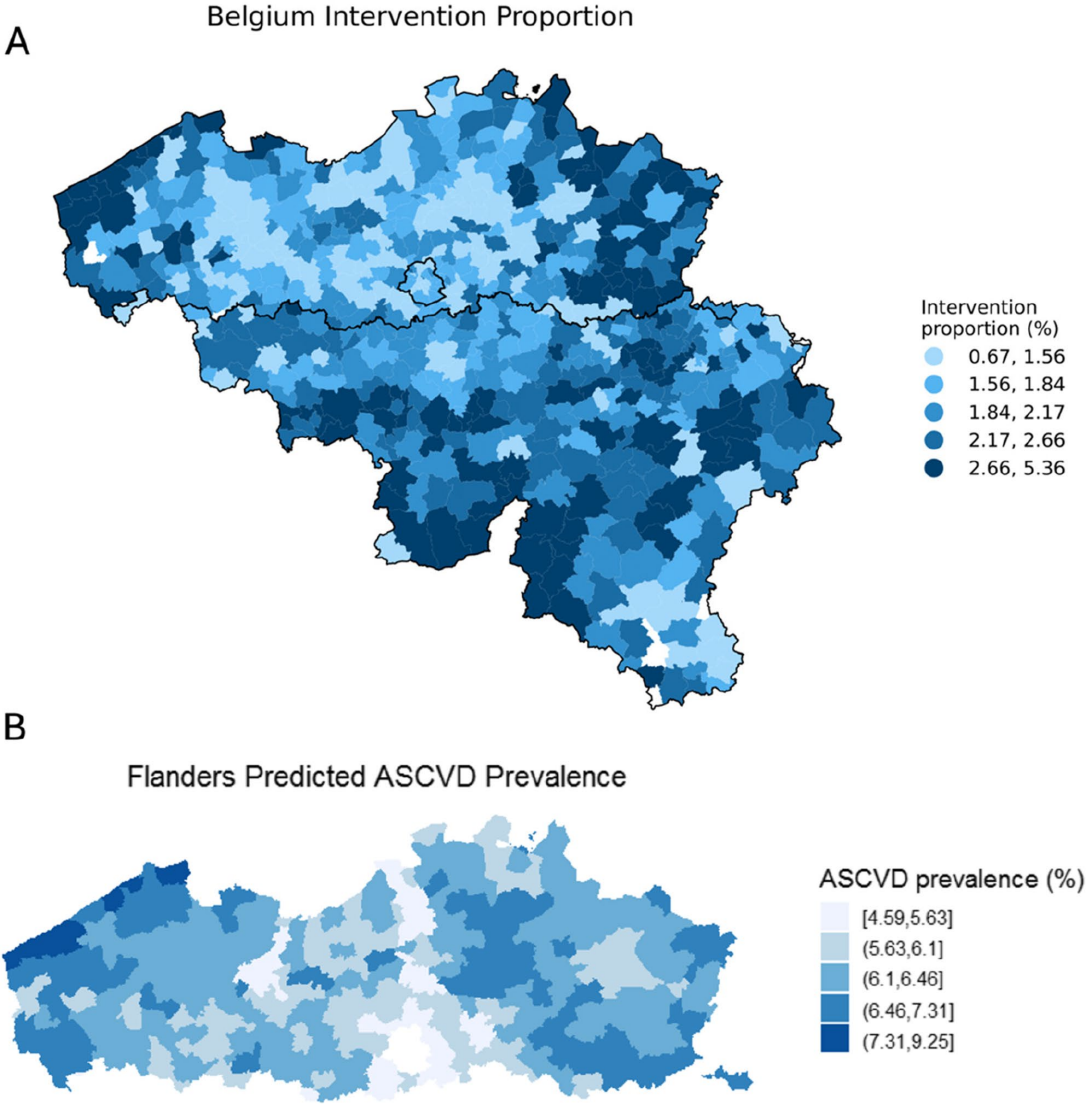
Geographic distribution of ASCVD intervention proportion based on small-area claims data across Belgium, at municipality level (**A**) and predicted ASCVD prevalence for municipalities in Flanders, obtained from the GP dataset (**B**). Darker color corresponds to a higher proportion and with elevated ASCVD prevalence. The white area at the bottom center of map B represents the Brussels capital region, which is not included in the region of Flanders, and not covered by the GP dataset

Overall ASCVD prevalence in the GP registry data was 6.46% and differed markedly across subgroups: 13.86% in patients aged 50 or older versus 0.95% in those younger than 50, 7.71% in males vs. 5.37% in females, 9.79% in patients with increased compensation versus 5.87% in those without, and 24.47% in patients with prescribed medication versus 2.79% in those without (see Table 2). Figure 1 displays the predicted ASCVD prevalence in Flanders. Higher prevalence is observed in western, coastal, and eastern areas, while lower predicted prevalence occurs around Brussels (bottom center), Ghent (center west) and Antwerp (center north).

### Multidimensional socioeconomic vulnerability index

**Fig. 2:**
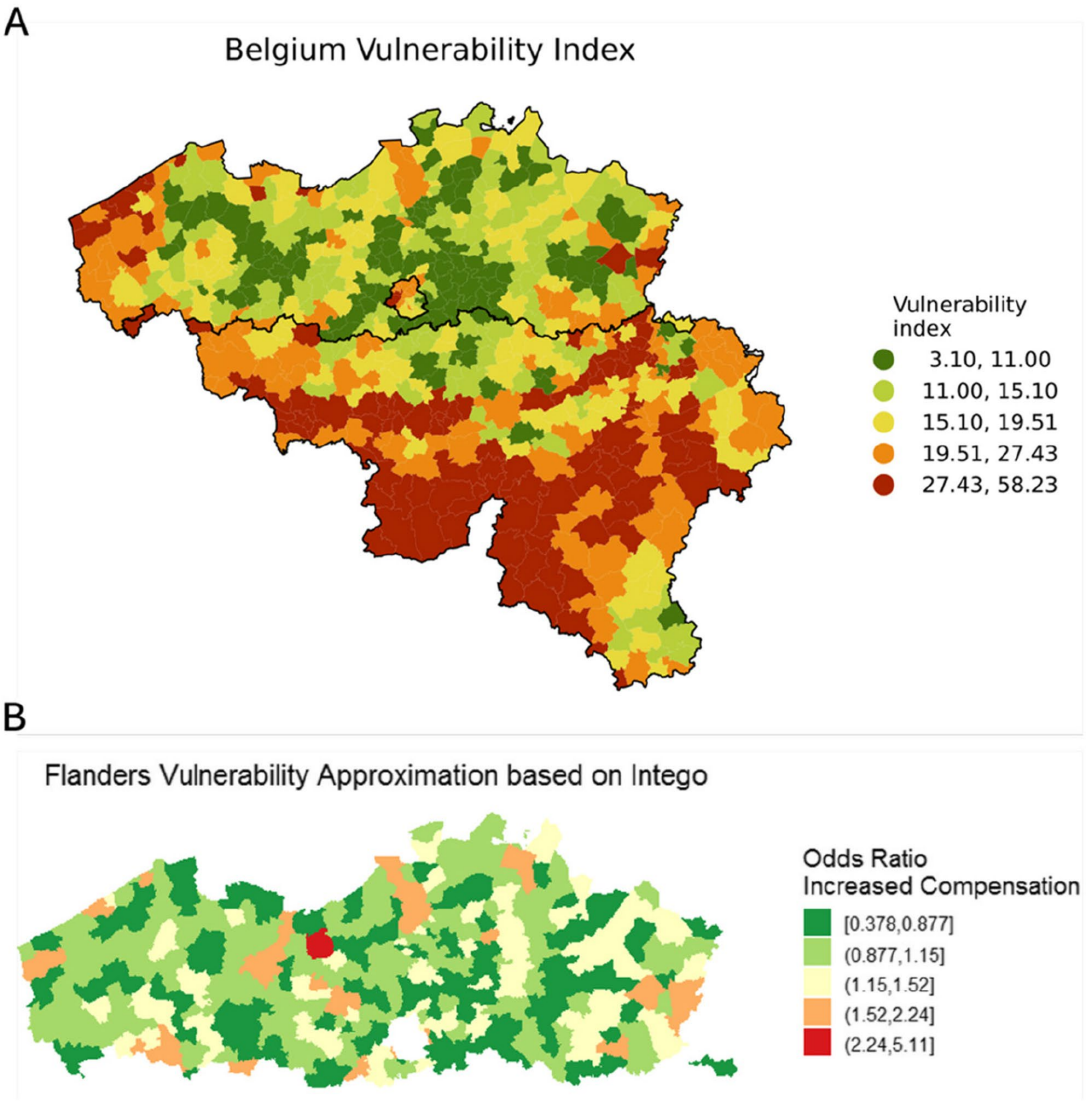
Geographic visualization of the multidimensional vulnerability index across Belgium at municipality level (**A**) and the municipality-level odds ratios of receiving increased compensation in Flanders based on the GP registry data (**B**). The color scale goes from dark green (low vulnerability) to yellow (medium vulnerability) to red (high vulnerability)

Figure 2 maps the vulnerability index at municipality-level across Belgium and the municipality-level odds ratios of receiving increased compensation in Flanders from the GP registry. More vulnerable regions are located near the coast (north-west), in the east, and south-west. Specifically for Flanders, the municipality-level proxy for vulnerability from the GP registry shows a moderate alignment with the vulnerability index (Spearman’s rho = 0.25, *p*-value < 0.001), with higher vulnerabilities in the east, Antwerp (north), three municipalities at the coast, and lower vulnerability around Brussels. Figure A6_2 in Additional file 6 additionally shows the upper and lower limits of the credible interval for the odds ratios.

### Link between ASCVD prevalence and socioeconomic vulnerability

#### Small-area claims data

Area-level vulnerability was positively correlated with indicators for ASCVD prevalence, including overall ASCVD interventions, use of lipid-lowering medications, and the proportions by corresponding strata based on increased compensation and age. ASCVD intervention was positively correlated with statin use, other lipid-lowering medication use, and their stratified versions (see Figure A8_1 in Additional file 8).

ASCVD prevalence indicators differed significantly between the most and least vulnerable municipalities, as shown in Table 3 (intervention proportion: U = 1614, *p* < 0.001; statin use proportion: U = 4504.5, *p* < 0.001). The most vulnerable municipalities had higher median values than the least vulnerable (intervention proportion: 2.7% vs. 1.72%; statin use proportion: 11.06% vs. 9.7%). Bootstrapping analysis showed that the median ASCVD intervention ratio was 1.57 times higher (95% CI: 1.46–1.65) in highest vulnerable municipalities compared with lowest vulnerable municipalities. Figure A8_2 in Additional file 8 displays the distributions of both indicators in the highest and lowest vulnerable municipalities.

**Table 3:** Comparison between ASCVD prevalence indicators in the most and least vulnerable municipalities (first and last quintiles)

Variables	Groups	*N*	Sum of Ranks	U test statistic	*P* value	Median (IQR)
ASCVD intervention	Most vulnerable municipalities	113	18048.00	1614.00	< 0.001	2.70 (0.95)
	Least vulnerable municipalities	117	8517.00			1.72 (0.48)
Statin	Most vulnerable municipalities	115	15620.50	4504.50	< 0.001	11.06 (2.89)
	Least vulnerable municipalities	117	11405.50			9.7 (2.55)

#### GP registry data

**Fig. 3:**
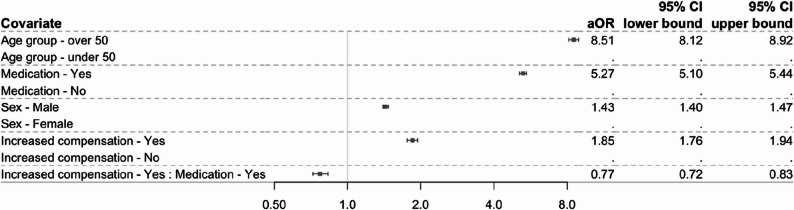
Forest plot of adjusted odds ratios with their 95% credible intervals from the GLMM

Municipality-level predicted ASCVD prevalence was positively correlated with municipality-level vulnerability (Spearman’s rho = 0.244, *p*-value < 0.001). Results from the Mann-Whitney U-test showed that the predicted ASCVD prevalence was higher in highly vulnerable municipalities compared to those with low vulnerabilities (*p*-value < 0.001). Figure A8_3 in Additional file 8 displays the distributions of the predicted ASCVD prevalence in the highest and lowest vulnerable municipalities. Bootstrapping analysis showed that the predicted ASCVD prevalence was 1.05 times higher (95% CI: 1.03–1.08) in high-vulnerability municipalities compared with low-vulnerability municipalities. Additionally, results showed statistically significant associations between individual-level factors and the prevalence of ASCVD (see Fig. 3). Males had higher odds of ASCVD (aOR = 1.43; 95% CI: 1.40–1.47) compared to females. Older individuals had over eight-fold increased odds of ASCVD (aOR = 8.51; 95% CI: 8.12–8.92) compared to younger ones. Increased compensation status and medication use were associated with higher odds of ASCVD, with a significant interaction effect (0.77; 95% CI: 0.72–0.83). For individuals without increased compensation, medication users had 5.27 (95% CI: 5.10–5.44) times the odds of ASCVD than non-users. Among individuals with increased compensation, medication use was linked with a 4.06 (0.77*5.27) increase in ASCVD odds. For individuals without medication, increased compensation related to a 1.85 (95% CI: 1.76–1.94) increase in ASCVD odds compared to those without increased compensation. For individuals using medication, the odds of ASCVD for those with increased compensation was 1.42 (0.77*1.85) times that of those without. The variance of the practice and the municipality random effects were 0.29 and 0.01, respectively, indicating a substantial variation between practices.

## Discussion

This study used a complementary, multi-level approach to examine ASCVD burden and socioeconomic vulnerability patterns in Belgium by analyzing two distinct datasets: small-area health insurance claims data and individual-level GP registry data, both providing surveillance insights at the municipality level. To enhance transparency, we developed a publicly accessible geoportal that visualizes geographic patterns of ASCVD and vulnerability, providing a novel resource for public health planning.

In 2022, approximately 750,000 Belgians were diagnosed with ASCVD [3]. Given a total population of 11,697,557 on January 1, 2023, this corresponds to an estimated national prevalence of 6.41%. This closely matched the 6.46% prevalence from GP registry data for Flanders but was higher than the 1.99% prevalence based on ASCVD intervention proportions from small-area claims data. These numbers reflect different definitions of ASCVD and levels of aggregation suitable for distinct surveillance purposes. Intervention-based estimates capture insured individuals undergoing ASCVD-related procedures, serving as a proxy for ASCVD prevalence, but also a proxy for healthcare utilization patterns at the municipality level. In contrast, the GP registry captures diagnosed ASCVD cases, including patients managed without intervention, and therefore provides a better estimate of ASCVD prevalence in Flanders. The lower intervention-based estimate likely reflects the fact that not all individuals with ASCVD receive a procedure. Together, these complementary indicators illustrate both the clinical and procedural dimensions of ASCVD surveillance in Flanders.

Our results revealed similar regional patterns of ASCVD prevalence across data sources, with higher values along the Belgian coast. This can largely be explained by demographic differences, as coastal municipalities tend to have a higher proportion of older residents, a key non-modifiable risk factor for ASCVD. The health insurance claims data also indicated clear north–south disparities, consistent with previous analyses of cardiovascular mortality [5]. The vulnerability index identified highly deprived areas near the border of Flanders and Wallonia, municipalities in East Flanders, coastal municipalities, and in the South-West of Belgium, broadly overlapping with ASCVD prevalence maps. These patterns were confirmed statistically, showing higher ASCVD prevalence in the most vulnerable quintiles, underscoring persistent health inequalities [22].

However, when stratified by increased compensation status, the small-area claims data showed a slightly lower median ASCVD prevalence among those with increased compensation (0.35%) compared to those without (1.68%). This finding contrasts with the individual-level GP registry data, where increased compensation was associated with higher odds of ASCVD. While lower prevalence in the increased compensation group seems unlikely, given established risk factor patterns [23], the discrepancy may reflect different aspects of ASCVD burden captured by each dataset. The claims-based ASCVD indicators may reflect healthcare utilization patterns rather than disease prevalence, potentially indicating differential access to ASCVD-related procedures among socially vulnerable populations [24].

To validate our small-area claims-based findings and enable municipality-level predictions, GP registry data confirmed well-established associations between ASCVD and non-modifiable risk factors, with males having higher odds than females, and individuals aged 50 or older showing substantially higher odds than those under 50 years [3]. Increased compensation status, serving as a proxy for socioeconomic vulnerability, was also associated with higher ASCVD prevalence. These individual-level patterns supported the feasibility of using GP data for area-level estimates of ASCVD burden. An interaction between socioeconomic vulnerability and medication use suggested that medication use may vary across socioeconomic groups. This finding points to potential differences in treatment access or adherence that could influence cardiovascular outcomes across socioeconomic groups. While lower SES patients typically show disadvantaged treatment patterns [24–26], our results showed that in the non-ASCVD population, they were more likely to receive lipid-lowering medication. This observation may reflect the GP registry’s focus on care-seeking individuals, where low SES patients may be diagnosed with other risk factors when consulting a GP, which may lead to prescriptions of lipid-lowering medication [27]. This also illustrates important considerations for interpreting surveillance data from GP registries.

A major strength of this study lies in its integration of small-area ASCVD data with a multidimensional socioeconomic vulnerability index. While previous studies have typically assessed individual indicators such as income, education, or employment in isolation [6–8], our approach captures the combined effects of multiple socioeconomic dimensions. The correlation between the vulnerability index and ASCVD indicators supports its validity and highlights its potential as a comprehensive measure of social vulnerability. Visualizing these data on a mapping platform enables identification of specific subnational regions with higher vulnerability and greater ASCVD burden, opening possibilities to guide geographically targeted public health interventions. The use of both small-area health insurance claims data and individual-level GP registry data provided complementary perspectives on ASCVD burden, strengthening the robustness and validity of observed associations across different healthcare contexts.

This study has several important limitations. Our observational, cross-sectional design prevents us from drawing causal inferences regarding the relationship between socioeconomic vulnerability and ASCVD burden, though establishing causality was not our primary objective. Rather, our goal was to provide insights into spatial inequalities in ASCVD and their association with socioeconomic vulnerability for public health surveillance. To achieve this, we analysed the two datasets independently and compared their findings. This deliberate approach allowed us to explore spatial inequalities using different sources rather than developing a unified analytical framework.

However, differences in sampling and selection biases between datasets may limit the generalizability of our findings. In the GP registry, estimated associations reflect relationships within the primary care-attending population and are therefore conditional on healthcare contact, which may not fully generalize to individuals who rarely consult primary care, despite the broadly representative demographic composition of the study population. At the same time, given that primary care serves as the first point of contact and coordinates chronic disease management in Flanders, modelling ASCVD burden based on GP registry data provides a clinically relevant approximation of population health needs within the healthcare system.

In the claims data, socioeconomically vulnerable individuals are underrepresented, and ASCVD indicators are based on cardiovascular interventions, which reflect healthcare utilization and access in addition to disease burden. Intervention rates were substantially lower among individuals receiving increased compensation, suggesting under-capture of ASCVD in vulnerable groups. However, because municipality-level information on the socioeconomic composition of MLOZ members is unavailable for this study and intervention-based indicators selectively capture treated cases, the direction and magnitude of bias in municipality-level associations with area-level vulnerability cannot be determined and warrant cautious interpretation. Nevertheless, the persistence of positive associations at the municipality level suggests that observed spatial patterns are robust and that they should be interpreted as conservative indicators of underlying socioeconomic inequalities.

Furthermore, GP registry data, while providing individual-level insights, cover only Flanders, which limits the generalizability of individual-level findings to the national level. Future research will focus on integrating these complementary data sources, taking into account their unique characteristics and strengths.

The multidimensional vulnerability index included a health domain, which introduces a degree of conceptual overlap with ASCVD outcomes. However, it has been shown that excluding the health domain from composite deprivation indices has minimal impact on area rankings and observed health inequalities [28]. The vulnerability measures also differed by analysis: area-level data used a multidimensional vulnerability index, while GP data relied on a binary proxy (increased compensation). To assess comparability, we compared the multidimensional index with area-level risk estimates for receiving increased compensation derived from GP data, examining how well these measures align spatially. Our ASCVD indicator, based on medication in the claims data, reflects treatment patterns rather than disease prevalence, creating a trade-off between sensitivity and specificity in identifying the target population. Additionally, it is important to note that, for some insights, we cannot make individual-level inferences from small-area claims data. Area-level associations between vulnerability and ASCVD indicators do not necessarily imply individual-level associations. Therefore, combining these insights with those from individual-level GP data registry strengthens our analysis. Privacy restrictions prevented access to absolute ASCVD counts, limiting direct prevalence estimation. Bootstrapping to estimate confidence intervals for predicted prevalence differences was performed on model predictions, not raw data, possibly underestimating true uncertainty. Finally, differences in healthcare-seeking behavior and access across socioeconomic groups may have led to underrepresentation of the most vulnerable populations.

## Conclusions

This study provides evidence suggestive of a consistent association between socioeconomic vulnerability and ASCVD prevalence in Belgium, observed across both individual-level clinical and small-area administrative data sources. The convergence of findings across two independent data sources with different methodological approaches, each with its distinct limitations, lends cautious confidence to the observed patterns. By integrating an area-level multidimensional vulnerability index with both data sources, we were able to identify regional disparities in cardiovascular health and detect regions of potentially elevated burden, particularly characterized by socioeconomic disadvantage. These spatial insights suggest a need for geographically targeted, area-level prevention and treatment strategies to improve cardiovascular outcomes in vulnerable communities. Disparities in lipid-lowering medication prescriptions further underscore the urgency of ensuring equitable access to preventive care and secondary prevention across socioeconomic groups [29].

Beyond its immediate findings, this study provides a preliminary framework for advancing equity-focused cardiovascular disease monitoring in Belgium. Combining small-area data, individual-level health data, and a multidimensional vulnerability index offers a scalable approach to identify and monitor health inequalities. Future research should build on this foundation by joint analyses of such datasets, and by integrating environmental and urban factors into this framework, which could further refine local public health strategies [30, 31]. Ultimately, such an integrative, data-driven approach can inform more precise and equitable policies to reduce the burden of ASCVD and promote cardiovascular health across diverse populations.

## Supplementary Information

Additional File 1: Study population demographics. Comparison tables between demographic distribution of the study populations and their respective general population.

Additional File 2: List of ASCVD interventions. List of ASCVD interventions used to extract the small-area health insurance claims data (MLOZ).

Additional File 3: List of ATC codes. List of ATC codes used to extract lipid-lowering medication indicators.

Additional File 4: List of ICPC-2 codes. List of diagnostic codes to extract clinical diagnosis of ASCVD from the GP registry.

Additional File 5: Definition of increased compensation in Flanders, Belgium. Definition of increased compensation in Flanders, Belgium.

Additional File 6: Model details for GP registry data. Details on the individual-level models used to (i) estimate associations, (ii) predict area-level ASCVD prevalence, and (iii) estimate risk of increased compensation from the individual-level GP registry data

Additional File 7: Domains and related datasets to compute the multidimensional vulnerability index.

Additional File 8: Additional figures on the area-level associations between ASCVD prevalence and socioeconomic vulnerability.

## Data Availability

The data underlying this article cannot be shared publicly due to confidentiality. The small-area health insurance claims data were provided by a third party, Multualités Libres - Onafhankelijke Ziekenfondsen (MLOZ), under licence / by permission. However, these data are visualized in an online dashboard, which can be consulted on https://ascvd-heatmap.epcon.ai. The individual-level GP data cannot be shared due to privacy reasons. This dataset contains real-life patient health data from electronic health records and stringent privacy measures are in place, making use of a data repository for open access impossible. Data are kept in a secure research environment. However, metadata on the database version used can be found on the website of KU Leuven RDR (https://doi.org/doi:10.48804/B1DB2X).

## Abbreviations

Abbreviation: Explanation
ASCVD: Atherosclerotic Cardiovascular Disease
ATC: Anatomical Therapeutic Chemical Classification System
BIMD: Belgian Index of Multiple Deprivation
CVD: Cardiovascular Disease
CI: Confidence Interval
GLMM: Generalized Linear Mixed Model
GP: General Practitioner
ICPC-2: International Codes of Primary Care, version 2
INLA: Intergrated Nested Laplace Approximation
MLOZ: Multualités Libres - Onafhankelijke Ziekenfondsen
SES: Socio Econmic Status
SM: Supplementary Material

## References

[1] World Health Organization. Cardiovascular diseases (CVDs). [cited 2025 Jun 18]. Available from: https://www.who.int/news-room/fact-sheets/detail/cardiovascular-diseases-(cvds).

[2] Vlayen J, De Backer G, Peers J, Moldenaers I, Debruyne H, Simoens S. Atherosclerotic Cardiovascular Diseases in Belgium: A Cost-of-illness Analysis. Cardiovasc Drugs Ther. 2008;22(6):487–94. 10.1007/s10557-008-6128-5. 18792772 10.1007/s10557-008-6128-5

[3] Maes I, Rey S. The status of atherosclerotic cardiovascular disease in Belgium, a silent and long-term killer: The prevalence, impact and cost of atherosclerotic cardiovascular disease (ASCVD) and recommendations for stakeholders to improve ASCVD in Belgium, 2022. Inovigate; 2022 Oct. Report No.

[4] Roth GA, Mensah GA, Johnson CO, Addolorato G, Ammirati E, Baddour LM, et al. Global Burden of Cardiovascular Diseases and Risk Factors, 1990–2019: Update From the GBD 2019 Study. J Am Coll Cardiol. 2020;76(25):2982–3021. 10.1016/j.jacc.2020.11.010. 33309175 10.1016/j.jacc.2020.11.010 PMC7755038

[5] Bevernaegie L, Devos I, Gadeyne S. Where did people die from cardiovascular disease? Spatial inequalities in cardiovascular mortality in Belgium between 1890 and 2011. Espace Popul Sociétés Space Popul Soc. 2024;(2023/3-2024/1):2023/3-2024/1. 10.4000/12tpu

[6] Brousmiche D, Lanier C, Cuny D, Frevent C, Genin M, Blanc-Garin C, et al. How do territorial characteristics affect spatial inequalities in the risk of coronary heart disease? Sci Total Environ. 2023;867:161563. 10.1016/j.scitotenv.2023.161563. 36640871 10.1016/j.scitotenv.2023.161563

[7] Kim E, Lee H, Lloyd-Jones D, Ko YG, Kim BG, Kim HC. Area deprivation and premature cardiovascular mortality: a nationwide population-based study in South Korea. BMJ Public Health. 2024;2(1). 10.1136/bmjph-2023-000877. PubMed PMID:. 10.1136/bmjph-2023-000877 PMC11812902 40018182

[8] Schultz WM, Kelli HM, Lisko JC, Varghese T, Shen J, Sandesara P, et al. Socioeconomic Status and Cardiovascular Outcomes: Challenges and Interventions. Circulation. 2018;137(20):2166–78. 10.1161/CIRCULATIONAHA.117.029652. PubMed PMID: 29760227; PubMed Central PMCID: PMC5958918. 29760227 10.1161/CIRCULATIONAHA.117.029652 PMC5958918

[9] Mousavi I, Suffredini J, Virani SS, Ballantyne CM, Michos ED, Misra A, et al. Early-onset atherosclerotic cardiovascular disease. Eur J Prev Cardiol. 2025;32(2):100–12. 10.1093/eurjpc/zwae240. 39041374 10.1093/eurjpc/zwae240

[10] World Health Organization. Social determinants of health. [cited 2025 Sep 4]. Available from: https://www.who.int/health-topics/social-determinants-of-health

[11] Xia M, An J, Safford MM, Colantonio LD, Sims M, Reynolds K, et al. Cardiovascular Risk Associated With Social Determinants of Health at Individual and Area Levels. JAMA Netw Open. 2024;7(4):e248584. 10.1001/jamanetworkopen.2024.8584. 38669015 10.1001/jamanetworkopen.2024.8584 PMC11053380

[12] Bensken WP, McGrath BM, Gold R, Cottrell EK. Area-level social determinants of health and individual-level social risks: Assessing predictive ability and biases in social risk screening. J Clin Transl Sci. 2023;7(1):e257. 10.1017/cts.2023.680. PubMed PMID: 38229891; PubMed Central PMCID: PMC10790234. 38229891 10.1017/cts.2023.680 PMC10790234

[13] Singh GK. Area Deprivation and Widening Inequalities in US Mortality, 1969–1998. Am J Public Health. 2003;93(7):1137–43. 10.2105/AJPH.93.7.1137 10.2105/ajph.93.7.1137 PMC1447923 12835199

[14] Flanagan BE, Gregory EW, Hallisey EJ, Heitgerd JL, Lewis B. A Social Vulnerability Index for Disaster Management. J Homel Secur Emerg Manag. 2011;8(1). 10.2202/1547-7355.1792.

[15] Jilani MH, Javed Z, Yahya T, Valero-Elizondo J, Khan SU, Kash B, et al. Social Determinants of Health and Cardiovascular Disease: Current State and Future Directions Towards Healthcare Equity. Curr Atheroscler Rep. 2021;23(9):55. 10.1007/s11883-021-00949-w. 34308497 10.1007/s11883-021-00949-w

[16] Otavova M, Masquelier B, Faes C, Van den Borre L, Bouland C, De Clercq E, et al. Measuring small-area level deprivation in Belgium: The Belgian Index of Multiple Deprivation. Spat Spatio-Temporal Epidemiol. 2023;45:100587. 10.1016/j.sste.2023.100587. 10.1016/j.sste.2023.100587 37301602

[17] Rey G, Jougla E, Fouillet A, Hémon D. Ecological association between a deprivation index and mortality in France over the period 1997–2001: variations with spatial scale, degree of urbanicity, age, gender and cause of death. BMC Public Health. 2009;9(1):33. 10.1186/1471-2458-9-33. 19161613 10.1186/1471-2458-9-33 PMC2637240

[18] Caspers M, Janssens A, Zayed A, Raat W, Van Pottelbergh G, Vaes B, Intego. -II database December 2024. KU Leuven RDR; 2025 [cited 2025 Apr 10]. Available from: https://rdr.kuleuven.be/dataset.xhtml?persistentId=doi:10.48804/B1DB2X doi:10.48804/B1DB2X.

[19] Statbel. Statistical sector update 2024. [cited 2025 Sep 4]. Available from: https://statbel.fgov.be/en/news/statistical-sector-update-2024

[20] Delvaux N, Aertgeerts B, van Bussel JC, Goderis G, Vaes B, Vermandere M. Health Data for Research Through a Nationwide Privacy-Proof System in Belgium: Design and Implementation. JMIR Med Inf. 2018;6(4):e11428. doi:10.2196/11428 PubMed PMID: 30455164; PubMed Central PMCID: PMC6300317. 10.2196/11428 PMC6300317 30455164

[21] Organisatie van zorg bij. chronische medicatie | NHG-Richtlijnen. [cited 2025 Sep 9]. Available from: https://richtlijnen.nhg.org/landelijke-eerstelijns-samenwerkingsafspraken/organisatie-van-zorg-bij-chronische-medicatie

[22] Aerts R, Nemery B, Bauwelinck M, Trabelsi S, Deboosere P, Van Nieuwenhuyse A, et al. Residential green space, air pollution, socioeconomic deprivation and cardiovascular medication sales in Belgium: A nationwide ecological study. Sci Total Environ. 2020;712:136426. 10.1016/j.scitotenv.2019.136426. PubMed PMID: 31945528. 31945528 10.1016/j.scitotenv.2019.136426

[23] Streel S, Donneau AF, Hoge A, Majerus S, Kolh P, Chapelle JP, et al. Socioeconomic Impact on the Prevalence of Cardiovascular Risk Factors in Wallonia, Belgium: A Population-Based Study. BioMed Res Int. 2015;2015:580849. 10.1155/2015/580849. PubMed PMID: 26380280; PubMed Central PMCID: PMC4561934. 26380280 10.1155/2015/580849 PMC4561934

[24] Hyun KK, Brieger D, Woodward M, Richtering S, Redfern J. The effect of socioeconomic disadvantage on prescription of guideline-recommended medications for patients with acute coronary syndrome: systematic review and meta-analysis. Int J Equity Health. 2017;16(1):162. 10.1186/s12939-017-0658-z. 28859658 10.1186/s12939-017-0658-z PMC5579970

[25] Devareddy A, Sarraju A, Rodriguez F. Health Disparities Across the Continuum of ASCVD Risk. Curr Cardiol Rep. 2022;24(9):1129–37. 10.1007/s11886-022. 01736-y PubMed PMID: 35788894; PubMed Central PMCID: PMC9378532. 35788894 10.1007/s11886-022-01736-y PMC9378532

[26] Ohm J, Skoglund PH, Häbel H, Sundström J, Hambraeus K, Jernberg T, et al. Association of Socioeconomic Status With Risk Factor Target Achievements and Use of Secondary Prevention After Myocardial Infarction. JAMA Netw Open. 2021;4(3):e211129. 10.1001/jamanetworkopen.2021.1129. 33688966 10.1001/jamanetworkopen.2021.1129 PMC7948055

[27] Leng B, Jin Y, Li G, Chen L, Jin N. Socioeconomic status and hypertension: a meta-analysis. J Hypertens. 2015;33(2):221. 10.1097/HJH.0000000000000428. 25479029 10.1097/HJH.0000000000000428

[28] McCartney G, Hoggett R, Walsh D, Lee D. How important is it to avoid indices of deprivation that include health variables in analyses of health inequalities? Public Health. 2023;221:175–80. 10.1016/j.puhe.2023.06.028. 37473649 10.1016/j.puhe.2023.06.028

[29] Bouckaert N, Maertens de Noordhout C, Van de Voorde C. Health System Performance Assessment: how equitable is the Belgian health system? [KCE Reports]. 2020. D/2020/10.273/30

[30] Dalton JE, Perzynski AT, Zidar DA, Rothberg MB, Coulton CJ, Milinovich AT, et al. Accuracy of Cardiovascular Risk Prediction Varies by Neighborhood Socioeconomic Position: A Retrospective Cohort Study. Ann Intern Med. 2017;167(7):456–64. 10.7326/M16-2543. PubMed PMID: 28847012; PubMed Central PMCID: PMC6435027. 28847012 10.7326/M16-2543 PMC6435027

[31] Tewma C, Mifsud JL. The impact of air pollution on atherosclerotic cardiovascular disease development. Br J Cardiol. 2024;31(2):013. 10.5837/bjc. 2024.013 PubMed PMID: 39555468; PubMed Central PMCID: PMC11562564. 39555468 10.5837/bjc.2024.013 PMC11562564

