# Supplementary material for: Spatial inequalities in cardiovascular health: a cross-sectional study with small-area health insurance claims and individual-level primary care data in Belgium
Source: BMC Public Health. 2026 Apr 23;26:1813. doi: 10.1186/s12889-026-27365-6 (PMC13244913; doi:10.1186/s12889-026-27365-6)
Supplement: Supplementary file 1 — Additional File 1: Study population demographics. Comparison tables between demographic distribution of the study populations and their respective general population. [file 12889_2026_27365_MOESM1_ESM.docx]

Additional file 1

Demographic distribution of the small-area claims data (MLOZ) and of the GP registry data (Intego) with respect to the general population of Belgium and Flanders, respectively.

# Table A1_1: demographic distribution of the small-area claims data (MLOZ) study population compared to the population of Belgium.

|  | **Belgium** | **Small-area claims** |
| --- | --- | --- |
| **Sex** |  |  |
| Male | 49% | 49% |
| Female | 51% | 51% |
| **Increased compensation** |  |  |
| Yes | 20% | 14% |
| No | 80% | 86% |
| **Age** |  |  |
| 0-9 year | 10% | 11% |
| 10-19 year | 12% | 13% |
| 20-29 year | 12% | 13% |
| 30-39 year | 13% | 14% |
| 40-49 year | 13% | 14% |
| 50-59 year | 13% | 14% |
| 60-69 year | 12% | 11% |
| 70-79 year | 9% | 7% |
| 80+ year | 6% | 4% |
| **Province** |  |  |
| Antwerpen | 16% | 14% |
| Brussels | 10% | 20% |
| Henegouwen | 11% | 11% |
| Limburg | 7% | 2% |
| Luik | 9% | 11% |
| Luxemburg | 2% | 1% |
| Namen | 4% | 4% |
| East-Flanders | 13% | 10% |
| Flemish-Brabant | 10% | 11% |
| Walloon-Brabant | 3% | 8% |
| West-Flanders | 10% | 6% |

# Figure A1_1: Geographical coverage of the small-area claims data (MLOZ) population on the level of arrondissement.


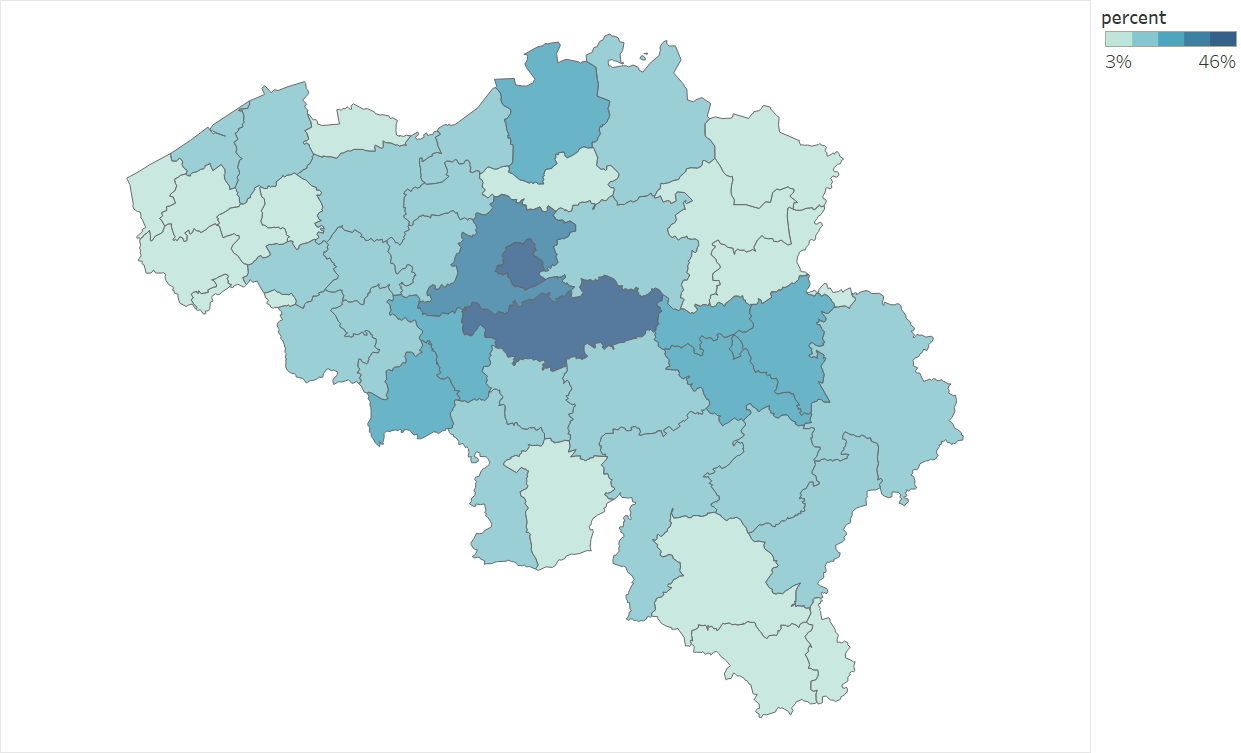


# Table A1_2: demographic distribution of the GP registry study (Intego) population compared to the population of Flanders.

|  | **Flanders** | **GP registry** |
| --- | --- | --- |
| **Sex** |  |  |
| Male | 50% | 47% |
| Female | 50% | 53% |
| **Increased compensation** |  |  |
| Yes | 17% | 15% |
| No | 83% | 85% |
| **Age** |  |  |
| 0-9 year | 10% | 9% |
| 10-19 year | 11% | 11% |
| 20-29 year | 11% | 11% |
| 30-39 year | 13% | 14% |
| 40-49 year | 13% | 13% |
| 50-59 year | 14% | 13% |
| 60-69 year | 13% | 13% |
| 70-79 year | 9% | 10% |
| 80+ year | 6% | 6% |
| **Province** |  |  |
| Antwerpen | 28% | 38% |
| Limburg | 13% | 12% |
| East-Flanders | 23% | 14% |
| Flemish-Brabant | 18% | 19% |
| West-Flanders | 18% | 17% |
