# Supplementary material for: Spatial inequalities in cardiovascular health: a cross-sectional study with small-area health insurance claims and individual-level primary care data in Belgium
Source: BMC Public Health. 2026 Apr 23;26:1813. doi: 10.1186/s12889-026-27365-6 (PMC13244913; doi:10.1186/s12889-026-27365-6)
Supplement: Supplementary file 2 — Additional File 2: List of ASCVD interventions. List of ASCVD interventions used to extract the small-area health insurance claims data (MLOZ). [file 12889_2026_27365_MOESM2_ESM.docx]

Additional file 2

List of ASCVD interventions used to extract the aggregated health insurance data (MLOZ).

| **Code** | **Description** |
| --- | --- |
| 158970 | All material for performing a percutaneous coronary intervention without stent |
| 158981 | All material for performing a percutaneous coronary intervention without stent |
| 158992 | All material for performing a percutaneous coronary intervention with placement of one or more stent (s) |
| 159003 | All material for performing a percutaneous coronary intervention with placement of one or more stent (s) |
| 159014 | All material for performing a percutaneous coronary intervention with placement of only one or more drug eluting stent (s) |
| 159025 | All material for performing a percutaneous coronary intervention with placement of only one or more drug eluting stent (s) |
| 159036 | All material for performing a percutaneous coronary intervention with the placement of only two or more drug Eluting Stents following the treatment of a multi -regessionate |
| 159040 | All material for performing a percutaneous coronary intervention with the placement of only two or more drug Eluting Stents following the treatment of a multi -regessionate |
| 160532 | All dilatation material and stent (s) used in revascularization of renal, mesenterial and supra aortic blood vessels, with the exception of the carotis blood vessels |
| 160532 | All dilatation material and stent (s) used in revascularization of renal, mesenterial and supra aortic blood vessels, with the exception of the carotis blood vessels |
| 160543 | All dilatation material and stent (s) used in revascularization of renal, mesenterial and supra aortic blood vessels, with the exception of the carotis blood vessels |
| 160543 | All dilatation material and stent (s) used in revascularization of renal, mesenterial and supra aortic blood vessels, with the exception of the carotis blood vessels |
| 160554 | All dilatation material and covered stent (s) used in revascularization of renal, mesenterial and supra aortic blood vessels, with the exception of the carotis blood vessels |
| 160554 | All dilatation material and covered stent (s) used in revascularization of renal, mesenterial and supra aortic blood vessels, with the exception of the carotis blood vessels |
| 160565 | All dilatation material and covered stent (s) used in revascularization of renal, mesenterial and supra aortic blood vessels, with the exception of the carotis blood vessels |
| 160565 | All dilatation material and covered stent (s) used in revascularization of renal, mesenterial and supra aortic blood vessels, with the exception of the carotis blood vessels |
| 160576 | All dilatation material and stent (s) used in revascularization of the lower limbs, aorto-iliacal, femoral, (infrastructure) Popliteal level |
| 160580 | All dilatation material and stent (s) used in revascularization of the lower limbs, aorto-iliacal, femoral, (infrastructure) Popliteal level |
| 160591 | All dilatation material and covered stent (s) used in revascularization of the lower limbs, aorto-iliacal, femoral, (infra) popliteal level |
| 160602 | All dilatation material and covered stent (s) used in revascularization of the lower limbs, aorto-iliacal, femoral, (infra) popliteal level |
| 160613 | All dilatation material and stent (s) used in revascularization of an artery on the contralateral side or of another anatomical axis, with the exception of the carotis blood vessels |
| 160613 | All dilatation material and stent (s) used in revascularization of an artery on the contralateral side or of another anatomical axis, with the exception of the carotis blood vessels |
| 160624 | All dilatation material and stent (s) used in revascularization of an artery on the contralateral side or of another anatomical axis, with the exception of the carotis blood vessels |
| 160624 | All dilatation material and stent (s) used in revascularization of an artery on the contralateral side or of another anatomical axis, with the exception of the carotis blood vessels |
| 160635 | All dilatation material and covered stent (s) used in revascularization of an artery on the contralateral side or of another anatomical axis, with the exception of the carotis blood vessels |
| 160635 | All dilatation material and covered stent (s) used in revascularization of an artery on the contralateral side or of another anatomical axis, with the exception of the carotis blood vessels |
| 160646 | All dilatation material and covered stent (s) used in revascularization of an artery on the contralateral side or of another anatomical axis, with the exception of the carotis blood vessels |
| 160646 | All dilatation material and covered stent (s) used in revascularization of an artery on the contralateral side or of another anatomical axis, with the exception of the carotis blood vessels |
| 160672 | All dilation material used for revascularization of renal, mesenteric, and supra-aortic blood vessels, excluding carotid blood vessels |
| 160683 | All dilation material used for revascularization of renal, mesenteric, and supra-aortic blood vessels, excluding carotid blood vessels |
| 160731 | All dilatation material used in revascularization of an artery on the contralateral side or of another anatomical axis, with the exception of the carotis blood vessels |
| 160742 | All dilatation material used in revascularization of an artery on the contralateral side or of another anatomical axis, with the exception of the carotis blood vessels |
| 160812 | Vascular stent used for femoro-popliteal bypass (above the knee) |
| 160823 | Vascular stent used for femoro-popliteal bypass (above the knee) |
| 160856 | Vascular stent used for femoro-distal bypass |
| 160860 | Vascular stent used for femoro-distal bypass |
| 160871 | Vascular stent used for Axilo-Femoral bypass |
| 160882 | Vascular stent used for Axilo-Femoral bypass |
| 160893 | Vascular stent used for aorta-iliac bypass |
| 160904 | Vascular stent used for aorta-iliac bypass |
| 160915 | Vascular stent used for aorta-femoral bypass |
| 160926 | Vascular stent used for aorta-femoral bypass |
| 160930 | Vascular stent used for Ilio-Femorale bypass |
| 160941 | Vascular stent used for Ilio-Femorale bypass |
| 160952 | Vascular stent used for thoracic bypass <15 cm |
| 160963 | Vascular stent used for thoracic bypass <15 cm |
| 160974 | Vascular stent used for thoracic bypass> and = 15 cm |
| 160985 | Vascular stent used for thoracic bypass> and = 15 cm |
| 161092 | Straight vascular stent used for vascular bypass, which is not defined in the provisions 160812-160823, 160834-160845, 160856-160860, 160871-160882, 160893-160904, 160915-160926, 160930-160941, 160952-160963, 160974-160985, 160996-161000, 161011-161022, 161033-161044, 161055-161066 en161070-161081 |
| 161103 | Straight vascular stent used for vascular bypass, which is not defined in the provisions 160812-160823, 160834-160845, 160856-160860, 160871-160882, 160893-160904, 160915-160926, 160930-160941, 160952-160963, 160974-160985, 160996-161000, 161011-161022, 161033-161044, 161055-161066 en161070-161081 |
| 170656 | Al material for performing a percutaneous coronary intervention without stent with one or more drug eluting balloons |
| 170660 | All material for performing a percutaneous coronary intervention without stent with one or more drug eluting balloons |
| 180235 | All dilatation material and one or more drug-coated balloon (NEN) used in revascularization of the superficial femoral artery and/or popliteal artery in combination with one or more stent (s) |
| 180246 | All dilatation material and one or more drug-coated balloon (NEN) used in revascularization of the superficial femoral artery and/or popliteal artery in combination with one or more stent (s) |
| 180250 | All dilatation material and one or more drug-coated balloon (NEN) used in revascularization of a superficial femoral artery and/or popliteal artery on the contralateral side, in combination with one or more stent (s) |
| 180261 | All dilatation material and one or more drug-coated balloon (NEN) used in revascularization of a superficial femoral artery and/or popliteal artery on the contralateral side, in combination with one or more stent (s) |
| 182011 | All dilatation material and drug-eluuting stent (s) used in revascularization of the femorale-popliteal arteries above the knee |
| 182022 | All dilatation material and drug-eluuting stent (s) used in revascularization of the femorale-popliteal arteries above the knee |
| 182033 | All dilatation material and drug-eluuting stent (s) used in revascularization of the femorale-popliteal arteries above the knee on the contralateral side |
| 182044 | All dilatation material and drug-eluuting stent (s) used in revascularization of the femorale-popliteal arteries above the knee on the contralateral side |
| 182136 | One or more thrombus retrievers used for an endovascular intracranial mechanical thrombectomy procedure for an ischemic cerebrovascular accident |
| 182140 | One or more thrombus retrievers used for an endovascular intracranial mechanical thrombectomy procedure for an ischemic cerebrovascular accident |
| 182151 | All catheterization material required for an endovascular removal of an intracranial thrombus via aspiration, or needed to use a Trombus retriever in an endovascular intracranial mechanical trombectomy procedure, in an ischemic cerebrovascular accident |
| 182162 | All catheterization material required for an endovascular removal of an intracranial thrombus via aspiration, or needed to use a Trombus retriever in an endovascular intracranial mechanical trombectomy procedure, in an ischemic cerebrovascular accident |
| 182173 | All of dilatation material and any stent used in an endovascular treatment of a proximally located arterial narrowing, to obtain access to an endovascular removal of an intracranial thrombus in an ischemic cerebrovascular accident |
| 182173 | All of dilatation material and any stent used in an endovascular treatment of a proximally located arterial narrowing, to obtain access to an endovascular removal of an intracranial thrombus in an ischemic cerebrovascular accident |
| 182184 | All of dilatation material and any stent used in an endovascular treatment of a proximally located arterial narrowing, to obtain access to an endovascular removal of an intracranial thrombus in an ischemic cerebrovascular accident |
| 182184 | All of dilatation material and any stent used in an endovascular treatment of a proximally located arterial narrowing, to obtain access to an endovascular removal of an intracranial thrombus in an ischemic cerebrovascular accident |
| 184693 | All of material required for the endovascular removal of a thrombus through aspiration and/or mechanical thrombectomy, in the case of arterial thrombosis |
| 184704 | All of material required for the endovascular removal of a thrombus through aspiration and/or mechanical thrombectomy, in the case of arterial thrombosis |
| 229095 | Revascularization of the myocardium through thoracotomy and direct intervention on the heart (Beck, O'Shaughnessy, Vineberg, etc.) |
| 229106 | Revascularization of the myocardium through thoracotomy and direct intervention on the heart (Beck, O'Shaughnessy, Vineberg, etc.) |
| 229574 | Myocardrevascularization by Anastomosis using the Arteria Mammaria Interna, with the use of the two arteria mammariae internae or implantation of the arteria mammaria interna in the form of sequential bridges |
| 229574 | Myocardrevascularization by Anastomosis using the Arteria Mammaria Interna, with the use of the two arteria mammariae internae or implantation of the arteria mammaria interna in the form of sequential bridges |
| 229585 | Myocardrevascularization by Anastomosis using the Arteria Mammaria Interna, with the use of the two arteria mammariae internae or implantation of the arteria mammaria interna in the form of sequential bridges |
| 229585 | Myocardrevascularization by Anastomosis using the Arteria Mammaria Interna, with the use of the two arteria mammariae internae or implantation of the arteria mammaria interna in the form of sequential bridges |
| 229611 | Myocardrevascularization carried out with an artery (breast collo, guest group cases or inflammatory artery) including any associated venous bypass (s) |
| 229611 | Myocardrevascularization carried out with an artery (breast collo, guest group cases or inflammatory artery) including any associated venous bypass (s) |
| 229622 | Myocardrevascularization carried out with an artery (breast collo, guest group cases or inflammatory artery) including any associated venous bypass (s) |
| 229622 | Myocardrevascularization carried out with an artery (breast collo, guest group cases or inflammatory artery) including any associated venous bypass (s) |
| 229633 | Myocardrevascularization on the beating heart carried out with an artery (breast colloica or inflated artery) including any associated venous bypass (s) |
| 229633 | Myocardrevascularization on the beating heart carried out with an artery (breast colloica or inflated artery) including any associated venous bypass (s) |
| 229644 | Myocardrevascularization on the beating heart carried out with an artery (breast colloica or inflated artery) including any associated venous bypass (s) |
| 229644 | Myocardrevascularization on the beating heart carried out with an artery (breast colloica or inflated artery) including any associated venous bypass (s) |
| 235071 | Revascularization of the Arteria carotis or vertebralis due to endarteriectomy, endoaneurysmorrhafie, pontage or resection with grafts or anastomosis |
| 235082 | Revascularization of the Arteria carotis or vertebralis due to endarteriectomy, endoaneurysmorrhafie, pontage or resection with grafts or anastomosis |
| 237171 | Unilateral aortofemoral, aorto-iliacal or iliacofemoral bridging |
| 237182 | Unilateral aortofemoral, aorto-iliacal or iliacofemoral bridging |
| 442676 | Positronentomographic examination with protocol and documents, for the whole of the research, when, in the case of a recently fully documented coronary insufficiency, a surgical procedure is provided, and there is still doubt about the viability of the Myocard |
| 442680 | Positronentomographic examination with protocol and documents, for the whole of the research, when, in the case of a recently fully documented coronary insufficiency, a surgical procedure is provided, and there is still doubt about the viability of the Myocard |
| 453596 | Digital Coronarography by heart catheterization with a minimum of two filmed gates per bridging |
| 453600 | Digital Coronarography by heart catheterization with a minimum of two filmed gates per bridging |
| 464192 | Digital Coronarography by heart catheterization with a minimum of two filmed gates per bridging |
| 464203 | Digital Coronarography by heart catheterization with a minimum of two filmed gates per bridging |
| 477724 | Honorarium for the physician specialist in neurology for the coordination of diagnostic opponiation and the preparation of a treatment plan by a multidisciplinary rowing team in the "intake" of a beneficiary who is admitted because of a recent cerebrovascular accident (CVA) |
| 477746 | Honorarium for the accredited physician specialist in neurology for the coordination of diagnostic opponiation and the preparation of a treatment plan by a multidisciplinary bore-up care team in the "intake" of a beneficiary who is included for a recent cerebrovascular accident (CVA) |
| 477761 | Honorarium for the physician specialist in neurology for the coordination of a multidisciplinary rowing team to draw up a care plan for a beneficiary who is included because of a cerebrovascular accident (CVA) |
| 477783 | Honorarium for the accredited physician specialist in neurology for the coordination of a multidisciplinary frosting team to draw up a care plan for a beneficiary who is included because of a cerebrovascular accident (CVA) |
| 588991 | Mechanical endovascular intracranial treatment of an acute ischemic cerebrovascular accident (thrombectomy), including manipulations and checks carried out during treatment and all consumption goods, with the exception of the catheters and medical devices required for removal of the clots and the fibrinolyticatica |
| 589002 | Mechanical endovascular intracranial treatment of an acute ischemic cerebrovascular accident (thrombectomy), including manipulations and checks carried out during treatment and all consumables, with the exception of the catheters and medical devices required for removal of the clots and the fibrinolyticatica |
| 589050 | Percutaneous endovascular dilatation with or without placement of stents (s) under control through medical imaging of an artiral narrowing and/or occlusion including manipulations and checks during treatment and all the equipment used, excluding the dilatation catheter (s), the pharmaca and and the contrast products. For the other arteries than the coronary |
| 589061 | Percutaneous endovascular dilatation with or without placement of stents (s) under control through medical imaging of an artiral narrowing and/or occlusion including manipulations and checks during treatment and all the equipment used, excluding the dilatation catheter (s), the pharmaca and and the contrast products. For the other arteries than the coronary |
| 590273 | Digital coronarography by heart catheterization with a minimum of two filmed sex excursions per bridge that is immediately followed by a percutaneous coronary intervention |
| 590284 | Digital coronarography by heart catheterization with a minimum of two filmed sex excursions per bridge that is immediately followed by a percutaneous coronary intervention |
| 680315 | SPANIES FOR MATERIAL FOR PERFORMATION A PERCUTANE Coronary intervention with placement of one or more drug Eluting Stent (s) Possibly in combination with one or more Bare Metal Stent (s) following the provision 589013-589024 within the indications provided in § 11ter |
| 680326 | SPANIES FOR MATERIAL FOR PERFORMATION A PERCUTANE Coronary intervention with placement of one or more drug Eluting Stent (s) Possibly in combination with one or more Bare Metal Stent (s) following the provision 589013-589024 within the indications provided in § 11ter |
| 680352 | Set of material for the performance of a percutaneous coronary intervention with placement of two or more stents following the handling of a multi-steldiease, either during the benefits 589013-589024 and 589035-589046, either during two benefits 589013-589024 at a different day |
| 680363 | Set of material for the performance of a percutaneous coronary intervention with placement of two or more stents following the handling of a multi-steldiease, either during the benefits 589013-589024 and 589035-589046, either during two benefits 589013-589024 at a different day |
| 683616 | All dilatation material and stent (s) used in revascularization of the lower limbs, aorto-iliacal, femoral, (infrastructure) Popliteal level |
| 683616 | All dilatation material and stent (s) used in revascularization of the lower limbs, aorto-iliacal, femoral, (infrastructure) Popliteal level |
| 683620 | All dilatation material and stent (s) used in revascularization of the lower limbs, aorto-iliacal, femoral, (infrastructure) Popliteal level |
| 683620 | All dilatation material and stent (s) used in revascularization of the lower limbs, aorto-iliacal, femoral, (infrastructure) Popliteal level |
| 683631 | All dilatation material and stent (s) used in revascularization of renal, mesenterial and supra aortic blood vessels, with the exception of the carotis blood vessels |
| 683631 | All dilatation material and stent (s) used in revascularization of renal, mesenterial and supra aortic blood vessels, with the exception of the carotis blood vessels |
| 683631 | All dilatation material and stent (s) used in revascularization of renal, mesenterial and supra aortic blood vessels, with the exception of the carotis blood vessels |
| 683642 | All dilatation material and stent (s) used in revascularization of renal, mesenterial and supra aortic blood vessels, with the exception of the carotis blood vessels |
| 683642 | All dilatation material and stent (s) used in revascularization of renal, mesenterial and supra aortic blood vessels, with the exception of the carotis blood vessels |
| 683642 | All dilatation material and stent (s) used in revascularization of renal, mesenterial and supra aortic blood vessels, with the exception of the carotis blood vessels |
| 683653 | All dilatation material and stent (s) used in revascularization of an artery on the contralateral side or of another anatomical axis, with the exception of the carotis blood vessels |
| 683653 | All dilatation material and stent (s) used in revascularization of an artery on the contralateral side or of another anatomical axis, with the exception of the carotis blood vessels |
| 683653 | All dilatation material and stent (s) used in revascularization of an artery on the contralateral side or of another anatomical axis, with the exception of the carotis blood vessels |
| 683664 | All dilatation material and stent (s) used in revascularization of an artery on the contralateral side or of another anatomical axis, with the exception of the carotis blood vessels |
| 683664 | All dilatation material and stent (s) used in revascularization of an artery on the contralateral side or of another anatomical axis, with the exception of the carotis blood vessels |
| 683664 | All dilatation material and stent (s) used in revascularization of an artery on the contralateral side or of another anatomical axis, with the exception of the carotis blood vessels |
| 683675 | All dilatation material and stent (s) used in venous revascularization |
| 683686 | All dilatation material and stent (s) used in venous revascularization |
| 683734 | All dilatation material used in revascularization of the lower limbs, aorto-iliacal, femoral, (infrastructure) Popliteal level |
| 683745 | All dilatation material used in revascularization of the lower limbs, aorto-iliacal, femoral, (infrastructure) Popliteal level |
| 683756 | All dilatation and revascularization material used for infrapopliteal revascularization for critical Limb Ischemia in patients with non-healing ulcerative wounds or nocturnal resting pain, with laser technology |
| 683760 | All dilatation and revascularization material used for infrapopliteal revascularization for critical Limb Ischemia in patients with non-healing ulcerative wounds or nocturnal resting pain, with laser technology |
| 683771 | All dilatation material used in revascularization of an artery on the contralateral side or of another anatomical axis, with the exception of the carotis blood vessels |
| 683782 | All dilatation material used in revascularization of an artery on the contralateral side or of another anatomical axis, with the exception of the carotis blood vessels |
| 687875 | All the way of material for performing a percutaneous coronary intervention with the placement of one or more stent (s) following the provision 589013 - 589024 |
| 687886 | All the way of material for performing a percutaneous coronary intervention with the placement of one or more stent (s) following the provision 589013 - 589024 |
| 687890 | SULCE OF MATERIAL For performing a percutaneous coronary intervention without stent following the provision 589013 - 589024 |
| 687901 | SULCE OF MATERIAL For performing a percutaneous coronary intervention without stent following the provision 589013 - 589024 |
| 715595 | All dilatation material and covered stent (s) used in revascularization of the lower limbs, aorto-iliacal, femoral, (infra) popliteal level |
| 715595 | All dilatation material and covered stent (s) used in revascularization of the lower limbs, aorto-iliacal, femoral, (infra) popliteal level |
| 715606 | All dilatation material and covered stent (s) used in revascularization of the lower limbs, aorto-iliacal, femoral, (infra) popliteal level |
| 715606 | All dilatation material and covered stent (s) used in revascularization of the lower limbs, aorto-iliacal, femoral, (infra) popliteal level |
| 715610 | All dilatation material and covered stent (s) used in revascularization of renal, mesenterial and supra aortic blood vessels, with the exception of the carotis blood vessels |
| 715610 | All dilatation material and covered stent (s) used in revascularization of renal, mesenterial and supra aortic blood vessels, with the exception of the carotis blood vessels |
| 715610 | All dilatation material and covered stent (s) used in revascularization of renal, mesenterial and supra aortic blood vessels, with the exception of the carotis blood vessels |
| 715621 | All dilatation material and covered stent (s) used in revascularization of renal, mesenterial and supra aortic blood vessels, with the exception of the carotis blood vessels |
| 715621 | All dilatation material and covered stent (s) used in revascularization of renal, mesenterial and supra aortic blood vessels, with the exception of the carotis blood vessels |
| 715621 | All dilatation material and covered stent (s) used in revascularization of renal, mesenterial and supra aortic blood vessels, with the exception of the carotis blood vessels |
| 715632 | All dilatation material and covered stent (s) used in revascularization of an artery on the contralateral side or of another anatomical axis, with the exception of the carotis blood vessels |
| 715632 | All dilatation material and covered stent (s) used in revascularization of an artery on the contralateral side or of another anatomical axis, with the exception of the carotis blood vessels |
| 715632 | All dilatation material and covered stent (s) used in revascularization of an artery on the contralateral side or of another anatomical axis, with the exception of the carotis blood vessels |
| 715643 | All dilatation material and covered stent (s) used in revascularization of an artery on the contralateral side or of another anatomical axis, with the exception of the carotis blood vessels |
| 715643 | All dilatation material and covered stent (s) used in revascularization of an artery on the contralateral side or of another anatomical axis, with the exception of the carotis blood vessels |
| 715643 | All dilatation material and covered stent (s) used in revascularization of an artery on the contralateral side or of another anatomical axis, with the exception of the carotis blood vessels |
| 715654 | All dilation material used for revascularization of renal, mesenteric, and supra-aortic blood vessels, excluding carotid blood vessels |
| 715665 | All dilation material used for revascularization of renal, mesenteric, and supra-aortic blood vessels, excluding carotid blood vessels |
| 715676 | Vascular stent used for femoro-popliteal bypass (above the knee) |
| 715680 | Vascular stent used for femoro-popliteal bypass (above the knee) |
| 715691 | Vascular stent used for femoro-femoral cross over |
| 715702 | Vascular stent used for femoro-femoral cross over |
| 715713 | Vascular stent used for femoro-distal bypass |
| 715724 | Vascular stent used for femoro-distal bypass |
| 715735 | Vascular stent used for Axilo-Femoral bypass |
| 715746 | Vascular stent used for Axilo-Femoral bypass |
| 715750 | Vascular stent used for aorta-iliac bypass |
| 715761 | Vascular stent used for aorta-iliac bypass |
| 715772 | Vascular stent used for aorta-femoral bypass |
| 715783 | Vascular stent used for aorta-femoral bypass |
| 715794 | Vascular stent used for Ilio-Femorale bypass |
| 715805 | Vascular stent used for Ilio-Femorale bypass |
| 715816 | Vascular stent used for thoracic bypass <15 cm |
| 715820 | Vascular stent used for thoracic bypass <15 cm |
| 715831 | Vascular stent used for thoracic bypass> and = 15 cm |
| 715842 | Vascular stent used for thoracic bypass> and = 15 cm |
| 715853 | Vascular type Valsalva with or without 1 side branch |
| 715864 | Vascular type Valsalva with or without 1 side branch |
| 715875 | Vascular stent with 1 or 2 side branches |
| 715886 | Vascular stent with 1 or 2 side branches |
| 715890 | Vascular stent with 3 or 4 side branches |
| 715901 | Vascular stent with 3 or 4 side branches |
| 715956 | Straight vascular stent used for vascular bypass, which is not defined in the provisions 715676-715680, 715691-715702, 715713-715724, 715735-715746, 715750-715761, 715772-715783, 715794-715805, 715816-715820, 715831-715842, 715853-715864, 715875-715886, 715890-715901, 715912-715923 en 715934-715945 |
| 715960 | Straight vascular stent used for vascular bypass, which is not defined in the provisions 715676-715680, 715691-715702, 715713-715724, 715735-715746, 715750-715761, 715772-715783, 715794-715805, 715816-715820, 715831-715842, 715853-715864, 715875-715886, 715890-715901, 715912-715923 en 715934-715945 |
| 798066 | Global prospective amount per admission in the context of a low -variable care stay: patient group (without ami or complex main diagnosis) that has undergone myocardial revascularization by means of a bypass, in classic hospitalization, with severity degrees 1 and 2 |
| We utilized the Nomensoft Riviz tool as the source terminology. The original descriptions were in Dutch and were translated into English using the GoogleTranslator function from the deep_translator library in Python. | |
