# Supplementary material for: Spatial inequalities in cardiovascular health: a cross-sectional study with small-area health insurance claims and individual-level primary care data in Belgium
Source: BMC Public Health. 2026 Apr 23;26:1813. doi: 10.1186/s12889-026-27365-6 (PMC13244913; doi:10.1186/s12889-026-27365-6)
Supplement: Supplementary file 3 — Additional File 3: List of ATC codes. List of ATC codes used to extract lipid-lowering medication indicators. [file 12889_2026_27365_MOESM3_ESM.docx]

Additional file 3

List of ATC codes

| **ATC code** | **substance** |
| --- | --- |
| C10AA01 | simvastatin; oral |
| C10AA03 | pravastatin; oral |
| C10AA04 | fluvastatin; oral |
| C10AA05 | atorvastatin; oral |
| C10AA07 | rosuvastatin; oral |
| C10AB02 | bezafibrate; oral |
| C10AB05 | fenofibrate; oral |
| C10AB08 | ciprofibrate; oral |
| C10AC01 | colestyramine; oral |
| C10AX06 | omega-3-triglycerides incl. other esters and acids; oral |
| C10AX09 | ezetimibe; oral |
| C10AX13 | evolocumab; parenteral |
| C10AX14 | alirocumab; parenteral |
| C10AX15 | bempedoic acid; oral |
| C10AX16 | inclisiran; systemic |
| C10BA02 | simvastatin and ezetimibe; oral |
| C10BA03 | pravastatin and fenofibrate; oral |
| C10BA05 | atorvastatin and ezetimibe; oral |
| C10BA06 | rosuvastatin and ezetimibe; oral |
| C10BA10 | bempedoic acid and ezetimibe; oral |
| C10BX06 | atorvastatin, acetylsalicylic acid and ramipril; systemic |
| C10BX11 | atorvastatin, aminopidine and perindpril; systemic |
| C10BX15 | atorvastatin and perindopril; systemic |
