# Supplementary material for: Spatial inequalities in cardiovascular health: a cross-sectional study with small-area health insurance claims and individual-level primary care data in Belgium
Source: BMC Public Health. 2026 Apr 23;26:1813. doi: 10.1186/s12889-026-27365-6 (PMC13244913; doi:10.1186/s12889-026-27365-6)
Supplement: Supplementary file 4 — Additional File 4: List of ICPC-2 codes. List of diagnostic codes to extract clinical diagnosis of ASCVD from the GP registry. [file 12889_2026_27365_MOESM4_ESM.docx]

Additional file 4

List of ASCVD diagnostic codes (ICPC-2) from Intego-II Primary Care Database (aka “the GP registry”).

| **ICPC-2 code** | **Description** |
| --- | --- |
| K74 | Ischaemic heart disease with angina |
| K75 | Acute myocardial infarction |
| K76 | Ischaemic heart disease without angina |
| K89 | Transient cerebral ischaemia |
| K90 | Stroke/cerebrovascular accident |
| K92 | Atherosclerosis/PVD |
