# Supplementary material for: Spatial inequalities in cardiovascular health: a cross-sectional study with small-area health insurance claims and individual-level primary care data in Belgium
Source: BMC Public Health. 2026 Apr 23;26:1813. doi: 10.1186/s12889-026-27365-6 (PMC13244913; doi:10.1186/s12889-026-27365-6)
Supplement: Supplementary file 5 — Additional File 5: Definition of increased compensation in Flanders, Belgium. Definition of increased compensation in Flanders, Belgium. [file 12889_2026_27365_MOESM5_ESM.docx]

Additional file 5

Definition of increased compensation in Flanders, Belgium.

Translated to English from <https://www.vlaanderen.be/verhoogde-tegemoetkoming-voor-gezondheidszorgen>

**Conditions – Increased Reimbursement (Verhoogde Tegemoetkoming)**

***Automatic entitlement (if you receive a social benefit)***

You automatically qualify for increased reimbursement if you receive one of the following benefits:

- Social integration income (leefloon) from the OCMW for at least 3 months
- Income guarantee for the elderly
- Allowance for persons with disabilities granted by the Federal Public Service - Social Security
- Allowance for assistance to the elderly granted by the Walloon Region or the Brussels-Capital Region
- Care budget for elderly persons with care needs granted by the Flemish Community

You also automatically qualify if:

- You are a child with a recognized disability of at least 66%
- You are an unaccompanied minor foreign national
- You are an orphan who has lost both parents

***If you do not automatically qualify, you can apply through your health insurance fund (ziekenfonds/mutualité).***

There are three possible situations:

1. You are single (with or without dependent children) and:

- Unemployed for at least 3 months
- Incapacitated for work for at least 3 months
- On long-term disability (invalidity)

1. You are in one of the following situations:

- Disabled or retired
- Officially recognized as a person with a disability
- Fully unemployed or incapacitated for at least 3 months
- Self-employed and receiving bridging rights for at least one quarter
- Single-parent family

1. You may still apply via your health insurance fund.

You must complete a declaration concerning your household income for the previous year.

That income must be below the annual threshold, calculated based on household size.

The fund will request supporting income documents, especially your latest tax assessment notice.
