# Supplementary material for: Spatial inequalities in cardiovascular health: a cross-sectional study with small-area health insurance claims and individual-level primary care data in Belgium
Source: BMC Public Health. 2026 Apr 23;26:1813. doi: 10.1186/s12889-026-27365-6 (PMC13244913; doi:10.1186/s12889-026-27365-6)
Supplement: Supplementary file 6 — Additional File 6: Model details for GP registry data. Details on the individual-level models used to (i) estimate associations, (ii) predict area-level ASCVD prevalence, and (iii) estimate risk of increased compensation from the individual-level GP registry data [file 12889_2026_27365_MOESM6_ESM.docx]

Additional file 6

Model details for GP registry data

### GP registry individual-level model formulation

We fitted a generalized linear mixed model (GLMM) with a logit link function for the GP registry data for two reasons: (i) to investigate the individual-level association between ASCVD and socioeconomic vulnerability and (ii) to predict municipality level ASCVD prevalence. This approach allowed adjustment for key confounders and accounted for the hierarchical structure of the data.

Let $Y_{i}$​ be a binary outcome variable indicating whether patient $i$, living in municipality *x_j_*, and visiting practice *z_k_,* was diagnosed with ASCVD (1 = yes, 0 = no). We assumed:

$Y_{ijk}\sim Bernoulli\left( p_{ijk} \right)$,

$logit\left( p_{ijk} \right)=log\left( \frac{p_{i,j,k}}{1-p_{i,j,k}} \right)= \mu_{i}+u(x_{j})+v(z_{k})$,

with $\mu_{i}$ containing the intercept and covariate effects,

$$\mu_{i}=\beta_{0}+\beta_{1}\cdot{AgeGroup}_{i}+\beta_{2}\cdot{Sex}_{i}+\beta_{3}\cdot{IncreasedCompensation}_{i}+$$

$$\beta_{4}\cdot{MedicationUse}_{i}+\beta_{5}\cdot{IncreasedCompensation\times{MedicationUse}_{i}}$$

where ${AgeGroup}_{i}$ binary indicator for patients aged $\geq50$ versus $<50$(reference), ${Gender}_{i}$ is a binary indicator for male versus female (reference), and ${IncreasedCompensation}_{i}$ is a binary indicator for receiving increased compensation (1=yes, 0=no). ${MedicationUse}_{i}$ is a binary indicator for being prescribed lipid-lowering medication (1=yes, 0=no). The interaction term ${IncreasedCompensation\times{MedicationUse}_{i}}$was found significant. $v(z_{k})$ and $u(x_{j})$ are random effects at the GP practice and municipality levels, respectively, to capture unobserved heterogeneity and clustering. In the final model, $v(z_{k})\sim N\left( 0,\sigma_{v}^{2} \right)$ and $u(x_{j})\sim N\left( 0,\sigma_{u}^{2} \right)$. This hierarchical model captures both practice-level and regional-level heterogeneity in ASCVD risk beyond what is explained by the fixed effects.

Models were fitted in R version 4.0.5 using the R-INLA package. The Integrated Nested Laplace Approximation (INLA) method was used for approximate Bayesian inference. We report results from the best-fitting model based on the Deviance Information Criterion (DIC) and Watanabe-Akaike Information Criterion (WAIC). After a prior sensitivity analysis, the default prior distributions in INLA were used for the precisions of the two random effects: $\tau\sim Gamma\left( 0,0.00005 \right).$

To determine the best-fitting model, we compared a range of candidate models using the DIC and the WAIC, with lower values indicating better model fit. Table A4_1 presents the DIC and WAIC values for all considered models. Among them, models 7, 8, and 9 showed the best performance. The DIC difference between model 8 and model 7 was less than five, suggesting a negligible difference in fit. Given this similarity and for the sake of interpretability and parsimony, we selected model 7 as the final model for further analysis. Model 7 includes sex, age group, increased compensation, medication use as binary variable, an interaction term between increased compensation and medication use, a Gaussian random effect at practice level and a Gaussian random effect at municipality level.

Table A6_1: Model comparison based on DIC and WAIC values. Models 7, 8, and 9 demonstrated the best performance, with the lowest DIC and WAIC. The difference in DIC and WAIC between models 8 and 7 were less than 5, indicating comparable model fit. For interpretability and parsimony, we selected model 7 as the final model.

| **Model ID** | **Model** | **DIC** | **WAIC** |
| --- | --- | --- | --- |
| 1 | fixed effects only | 28132.59 | 28154.66 |
| 2 | + practice (i.i.d.) | 23434.59 | 23502.91 |
| 3 | + practice (i.i.d) and municipality (i.i.d.) | 23392.08 | 23464.91 |
| 4 | + practice (i.i.d.) and municipality (CAR) | 23391.31 | 23463.38 |
| 5 | + practice (i.i.d.) and municipality (BYM2) | 23389.32 | 23462.75 |
| 6 | + interaction, practice (i.i.d.) | 23377.98 | 23442.93 |
| 7 | + interaction, practice (i.i.d.) and municipality (i.i.d.) | 23335.28 | 23404.66 |
| 8 | + interaction, practice (i.i.d.) and municipality (BYM2) | 23332.74 | 23402.48 |
| 9 | + interaction, practice (i.i.d.) and municipality (CAR) | 23334.90 | 23403.24 |
| Interaction: medication_use*increased_compensation; i.i.d.: independently and identically distributed practice effect; CAR: conditionally autoregressive spatial effect; BYM2: i.i.d. + CAR spatial effect at municipality level. | | | |

### Small-area ASCVD prevalence prediction from individual-level model

We predicted ASCVD prevalence in the municipalities of Flanders by using a logistic regression model fitted on the individual-level GP registry data. The model included age group (<50, ≥50), sex, increased compensation (as a proxy for socioeconomic vulnerability), and binary medication use, as well as an interaction term between increased compensation and medication use. Since medication use was not available in the publicly accessible population data, we computed the proportion of individuals receiving medication (i.e., the probability of medication use) for each stratum defined by age, sex, and increased compensation in the GP registry dataset. Public population data, including age, sex, and increased compensation distributions at the municipality level, were obtained from the IMA-Atlas (<https://ima-aim.be/IMA-Atlas?lang=nl>). These data were then used to predict ASCVD probabilities for each municipality by applying the fitted logistic regression model. Finally, these predictions were weighted by the estimated medication use proportions, resulting in an aggregated estimate of ASCVD prevalence per municipality. For this analysis, we focused on a model without random effects to facilitate prediction on population data.


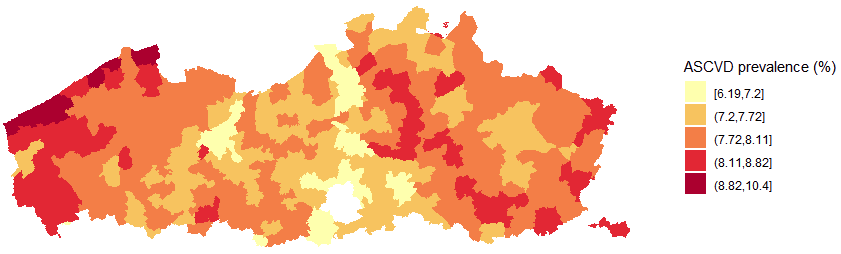


Figure A6_1: Predicted ASCVD prevalence for municipalities in Flanders was obtained by applying the model to public demographic data, weighting predictions based on medication use from the GP registry dataset. Darker colors indicate regions with elevated ASCVD prevalence. The white area at the bottom center of the map represents Brussels, which is outside Flanders.

### Relative risk of increased compensation

The vulnerability index is a composite measure of multiple deprivation, incorporating various socioeconomic variables. In the Intego database, socioeconomic status (SES) is approximated by the indicator of receiving increased compensation. This analysis aims to assess whether the SES indicator from Intego exhibits similar spatial patterns to the vulnerability index across Flanders. To this end, municipality-level odds ratios of receiving increased compensation were computed using a Binomial GLMM.

Let $Y_{i}$​ be a binary outcome variable indicating whether patient $i$, living in municipality *x_j_*, and visiting practice *z_k_,* received increased compensation (1 = yes, 0 = no). We assumed:

$Y_{ijk}\sim Bernoulli\left( p_{ijk} \right)$,

$logit\left( p_{ijk} \right)=log\left( \frac{p_{i,j,k}}{1-p_{i,j,k}} \right)= \mu_{i}+u(x_{j})+v(z_{k})$,

with $\mu_{i}$ containing the intercept and covariate effects,

$$\mu_{i}=\beta_{0}+\beta_{1}\cdot{AgeGroup}_{i}+\beta_{2}\cdot{Sex}_{i}$$

where ${AgeGroup}_{i}$ binary indicator for patients aged $\geq50$ versus $<50$(reference), ${Gender}_{i}$ is a binary indicator for male versus female (reference). $v(z_{k})$ and $u(x_{j})$ are random effects at the GP practice and municipality levels, respectively, to capture unobserved heterogeneity and clustering. In the final model, $v(z_{k})\sim N\left( 0,\sigma_{v}^{2} \right)$ and $u(x_{j})\sim N\left( 0,\sigma_{u}^{2} \right)$. This hierarchical model captures both practice-level and regional-level heterogeneity in increased compensation risk beyond what is explained by the fixed effects.

Models were fitted in R version 4.0.5 using the R-INLA package. The Integrated Nested Laplace Approximation (INLA) method was used for approximate Bayesian inference. We report results from the best-fitting model based on the Deviance Information Criterion (DIC) and Watanabe-Akaike Information Criterion (WAIC). Default prior distributions in INLA were used for the precisions of the two random effects: $\tau\sim Gamma\left( 0,0.00005 \right).$

To determine the best-fitting model, we compared a range of candidate models using the DIC and the WAIC, with lower values indicating better model fit. Table SM3 presents the DIC and WAIC values for all considered models. Among them, models 3 and 5 showed the best performance. The DIC difference between model 3 and model 5 was less than five, suggesting a negligible difference in fit. Given this similarity and for the sake of interpretability and parsimony, we selected model 3 as the final model for further analysis. Model 3 includes sex and age group, a Gaussian random effect at practice level and a Gaussian random effect at municipality level.

Table A6_2: Model comparison based on DIC and WAIC values. Models 3 and 5 demonstrated the best performance, with the lowest DIC and WAIC. The difference in DIC and WAIC between models 3 and 5 were less than 5, indicating comparable model fit. For interpretability and parsimony, we selected model 3 as the final model.

| **Model ID** | **Model** | **DIC** | **WAIC** |
| --- | --- | --- | --- |
| 1 | fixed effects only | 51681.93 | 52343.34 |
| 2 | + practice (i.i.d.) | 27636.18 | 28598.10 |
| 3 | + practice (i.i.d) and municipality (i.i.d.) | 25973.48 | 26869.80 |
| 4 | + practice (i.i.d.) and municipality (CAR) | 25984.66 | 26882.93 |
| 5 | + practice (i.i.d.) and municipality (BYM2) | 25973.20 | 26869.53 |
| i.i.d.: independently and identically distributed practice effect; CAR: conditionally autoregressive spatial effect; BYM2: i.i.d. + CAR spatial effect at municipality level. | | | |

Figure A6_2 shows the map of the estimated odds ratios. Similar to the map of the vulnerability index, we observe higher odds of receiving increased compensation, or higher vulnerability, in the east of Limburg and near the coast, while lower odds, or lower vulnerability in the region around Brussels. The spearman’s rank correlation rho between the municipality level relative risk estimates and vulnerability index was 0.25 and statistically significant (p < 0.001). These results suggest that municipalities with higher relative risks of increased compensation also tend to exhibit higher values on the vulnerability index map. This overlap supports our hypothesis that the indicator of increased compensation in the GP registry database reflects underlying socioeconomic vulnerability.


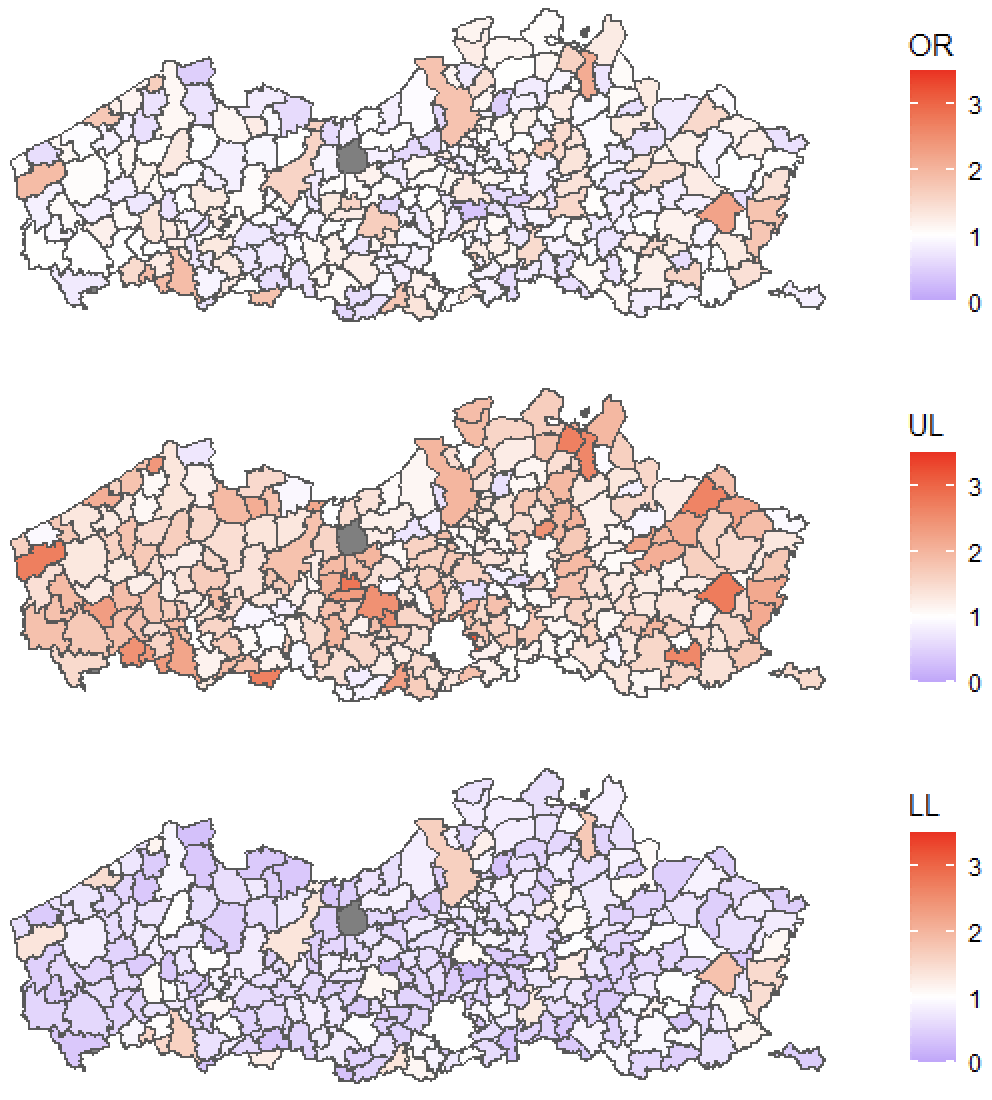


Figure A6_2: Odds Ratio (OR; top) of receiving increased compensation across municipalities in Flanders, along with the upper (UL; middle) and lower (LL; bottom) bounds of the 95% credible interval. Estimates are based on a Binomial GLMM accounting for age, sex, and practice.
