# Supplementary material for: Spatial inequalities in cardiovascular health: a cross-sectional study with small-area health insurance claims and individual-level primary care data in Belgium
Source: BMC Public Health. 2026 Apr 23;26:1813. doi: 10.1186/s12889-026-27365-6 (PMC13244913; doi:10.1186/s12889-026-27365-6)
Supplement: Supplementary file 7 — Additional File 7: Domains and related datasets to compute the multidimensional vulnerability index. [file 12889_2026_27365_MOESM7_ESM.docx]

Additional file 7

Domains and related datasets to compute the multidimensional vulnerability index

| **Domain** | **Variable** | **Source** | **Year** | **Spatial resolution** | **Definition** | **Variables in BIMD,2023** |
| --- | --- | --- | --- | --- | --- | --- |
| Income | MAF receivers | IMA | 2021 | Statistical sector | Percentage of beneficiaries who have exceeded their MAF threshold and can therefore receive a MAF allowance. | "Proportion of individuals in the lowest income decile  Proportion of households with net taxable income  below the minimum living wage" |
| Income | Taxable income | IMA | 2019 | Statistical sector | Median taxable income (individual and joint returns). |  |
| Income | Interquartile coefficient | Statbel | 2021 | Statistical sector | The difference between the 3rd and 1st quartiles relative to the median: (Q3-Q1)/Q2. The higher the interquartile coefficient, the higher the degree of income inequality. |  |
| Employment | Unemployed | Census | 2021 | Statistical sector | Number of unemployed compared to active (employed or unemployed). | "Proportion of working age population who is  unemployed  Proportion of working age population who is not  working due to disability  " |
| Employment | Replacement income | IMA | 2021 | Statistical sector | Percentage of beneficiaries with at least 250 days of replacement income (age 20-64, no pensioner status, holder). |  |
| Employment | Long-term disability benefit | IMA | 2021 | Statistical sector | Percentage of beneficiaries with at least 120 days of disability benefits (age 20-64, no pensioner status, holder). |  |
| Education | Upper secondary education at most | Census | 2021 | Statistical sector | Number of population with maximum higher secondary education divided by population. | "Proportion of early school leavers  NEET indicator  Proportion of working age adults without qualification" |
| Education | Higher education men | Census | 2021 | Municipality | Percentage of men aged 25-64 with a higher education diploma. |  |
| Education | Higher education women | Census | 2021 | Municipality | Percentage of women aged 25-64 with a higher education diploma. |  |
| Housing | Proportion tenants/owners | Census | 2021 | Statistical sector | Proportion of population of rental properties/owner-occupied properties. | "Proportion of tenants  Proportion of individuals living in dwellings:  smaller than 35 m2  less than 0.5 room/person  without central heating  without insulation  without kitchen  without toilet  without bathroom  without internet" |
| Housing | No central heating | Census | 2021 | Statistical sector | Number of houses without central heating divided by total houses. |  |
| Housing | Less then 1 living room per resident | Census | 2021 | Statistical sector | Number of houses with 0.5 to less than 1 living room divided by total houses. |  |
| Health | Mortality rate | Statbel | 2023 | Municipality | Number of deaths divided by population. | "Standardized mortality ratio  Standardized suicide rate  Preventable mortality fraction" |
| Health | Antidepressiva users | IMA | 2022 | Statistical sector | Percentage of antidepressant users per calendar year. |  |
| Health | Prevalence Diabetes | IMA | 2022 | Statistical sector | Number of beneficiaries with antidiabetic drug deliveries or with nomenclature referring to diabetes per 1,000 beneficiaries. |  |
| Health | Prevalence polypharmacy | IMA | 2022 | Statistical sector | The number of beneficiaries per 1000 who chronically (≥ 80 DDD) use 5 or more reimbursed medicines (at ATC-5 level) by sex, age category, VT and type of long-term care. |  |
| Crime | Property crimes | Federal Police Belgium | 2023 | Municipality | Number of facts registered by the police services for the selected crime divided by population. | "Rate of property crimes  Rate of violent crimes  Rate of family violence crimes  Rate of fraud " |
| Crime | Violent crimes | Federal Police Belgium | 2023 | Municipality | Number of facts registered by the police services for the selected crime divided by population. |  |
| Crime | Domestic violence crimes | Federal Police Belgium | 2023 | Municipality | Number of facts registered by the police services for the selected crime divided by population. |  |
| Crime | Drug crimes | Federal Police Belgium | 2023 | Municipality | Number of facts registered by the police services for the selected crime divided by population. |  |
| Crime | Fraude | Federal Police Belgium | 2023 | Municipality | Number of facts registered by the police services for the selected crime divided by population. |  |
