# Supplementary material for: Spatial inequalities in cardiovascular health: a cross-sectional study with small-area health insurance claims and individual-level primary care data in Belgium
Source: BMC Public Health. 2026 Apr 23;26:1813. doi: 10.1186/s12889-026-27365-6 (PMC13244913; doi:10.1186/s12889-026-27365-6)
Supplement: Supplementary file 8 — Additional File 8: Additional figures on the area-level associations between ASCVD prevalence and socioeconomic vulnerability. [file 12889_2026_27365_MOESM8_ESM.docx]

Additional file 8

Additional figures on the area-level associations between ASCVD prevalence and socioeconomic vulnerability

### Figure A8_1: One-to-one Spearman’s correlations between vulnerability, demography, ASCVD interventions, and lipid-lowering therapy.


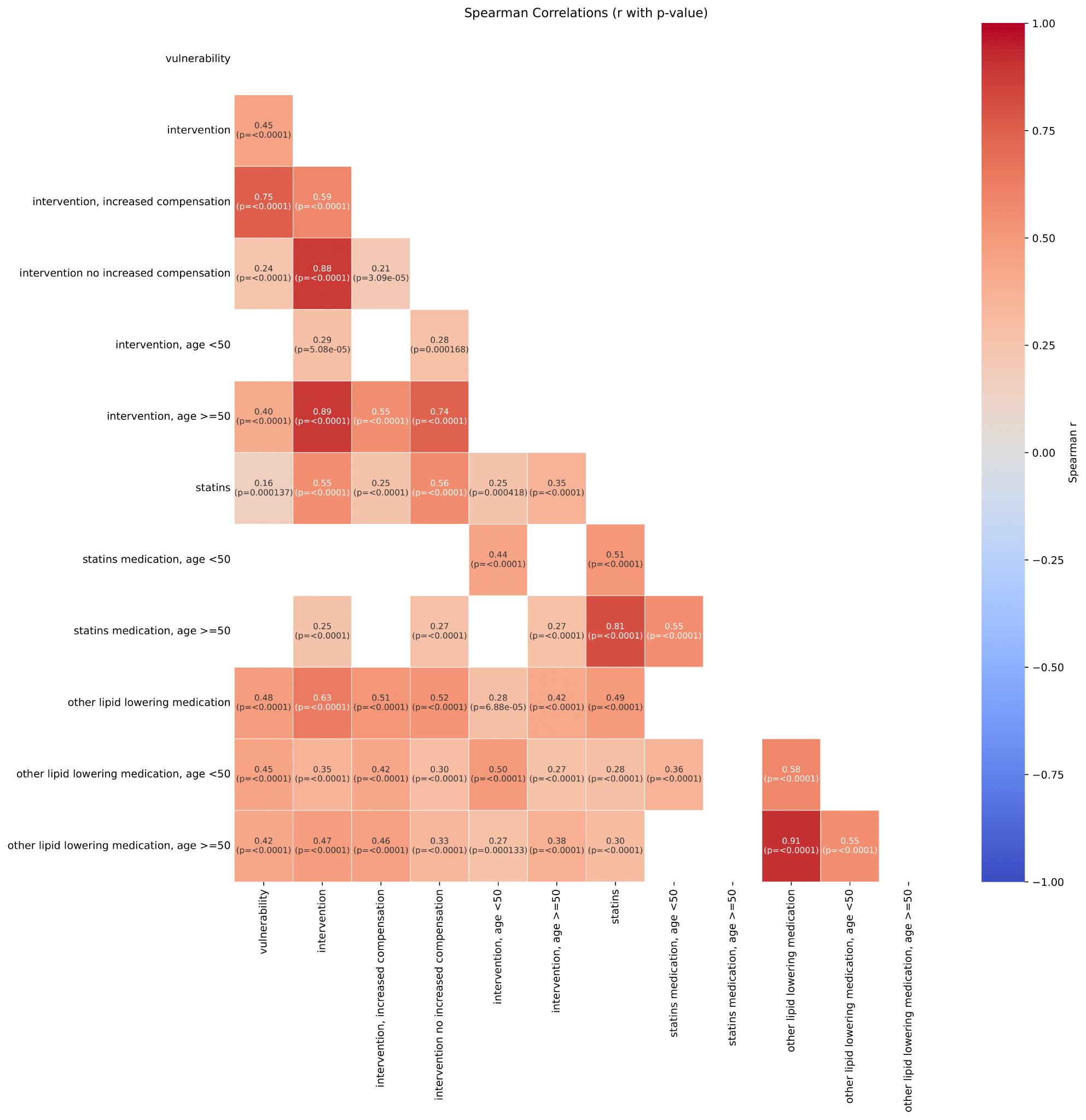


### Figure A8_2: Boxplots of ASCVD prevalence indicators of the aggregated health insurance funds in highest and lowest vulnerable municipalities


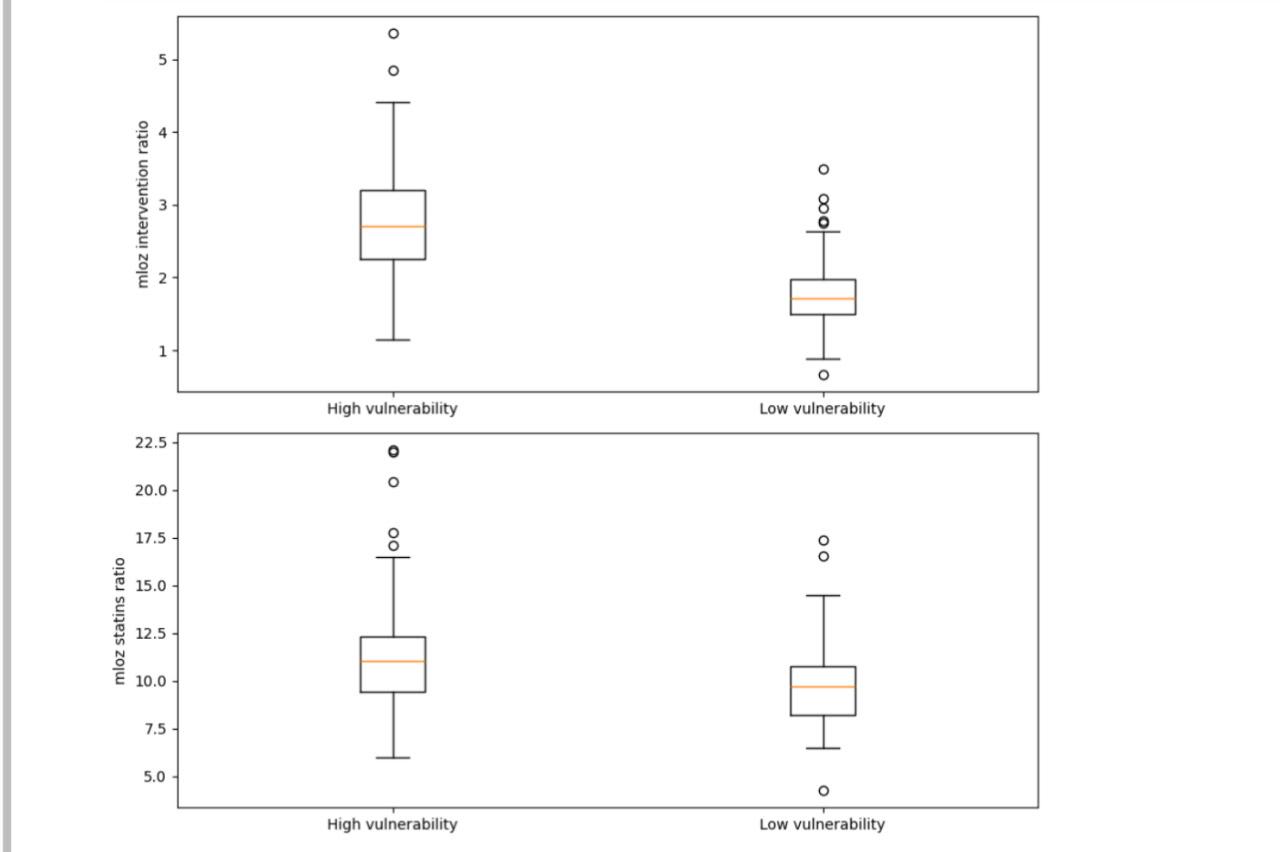


Figure A8_2: Graphical representation of distribution of the ASCVD prevalence indicators in the most (high) and least (low) vulnerable municipalities (first and last quintiles).

### Figure A8_3: Boxplot of predicted ASCVD prevalence from the GP registry data in highest and lowest vulnerable municipalities


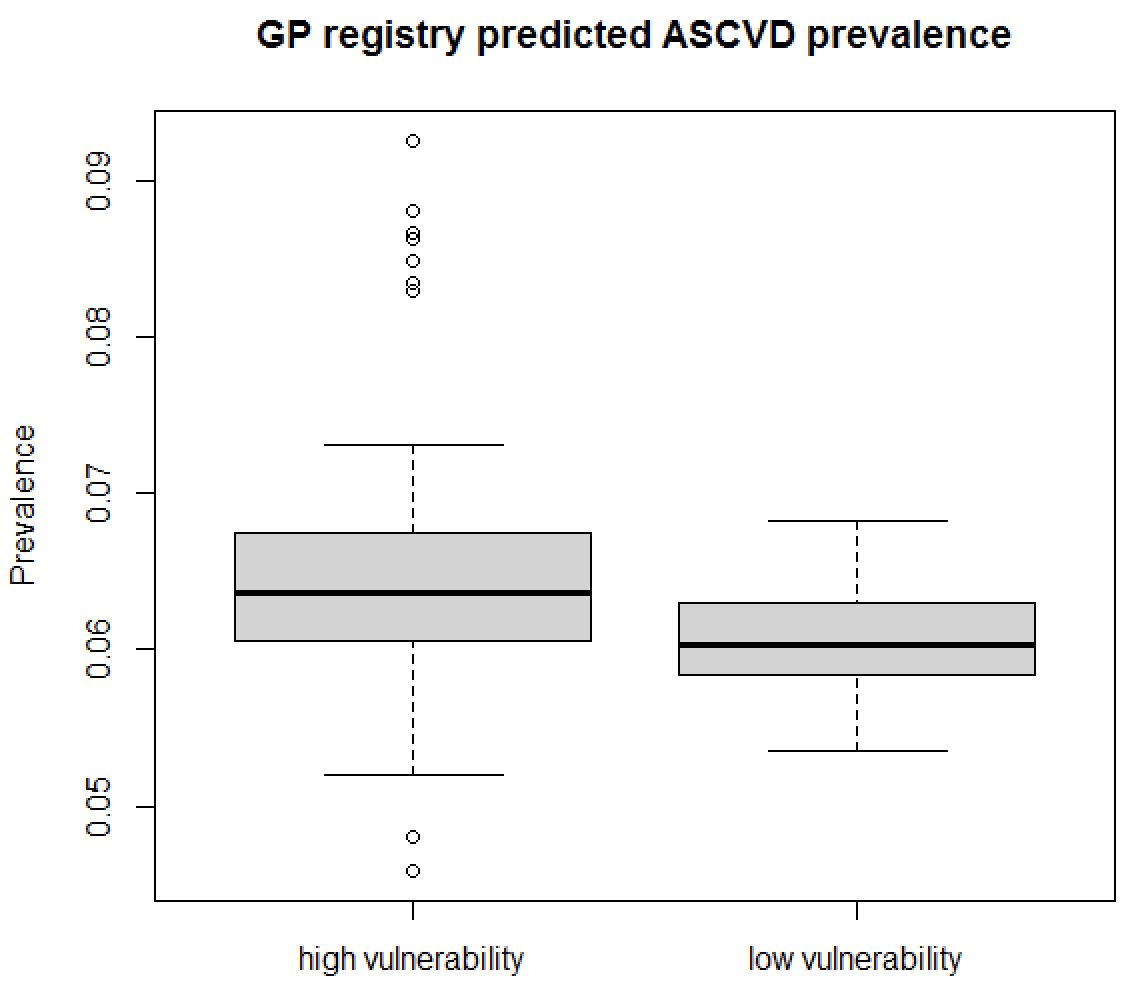


Figure A8_3: Graphical representation of distribution of the predicted ASCVD prevalence in the most (high) and least (low) vulnerable municipalities (first and last quintiles).
